# RGD Surface Functionalization of the Hydrophilic Acrylic Intraocular Lens Material to Control Posterior Capsular Opacification

**DOI:** 10.1371/journal.pone.0114973

**Published:** 2014-12-11

**Authors:** Yi-Shiang Huang, Virginie Bertrand, Dimitriya Bozukova, Christophe Pagnoulle, Christine Labrugère, Edwin De Pauw, Marie-Claire De Pauw-Gillet, Marie-Christine Durrieu

**Affiliations:** 1 Departments of Chemistry & Bio-Medical and Preclinical Sciences, Mass Spectrometry Laboratory & Mammalian Cell Culture Laboratory – GIGA R, Université de Liège, Liège, Belgium; 2 CBMN UMR5248, Institute of Chemistry & Biology of Membranes & Nanoobjects, Université de Bordeaux, Pessac, France; 3 Research and Development Department, PhysIOL SA, Liège, Belgium; 4 PLACAMAT, Plateforme Aquitaine de Caractérisation des Matériaux, UMS 3626, Université de Bordeaux, Pessac, France; Université de Technologie de Compiègne, France

## Abstract

Posterior Capsular Opacification (PCO) is the capsule fibrosis developed on implanted IntraOcular Lens (IOL) by the de-differentiation of Lens Epithelial Cells (LECs) undergoing Epithelial Mesenchymal Transition (EMT). Literature has shown that the incidence of PCO is multifactorial including the patient's age or disease, surgical technique, and IOL design and material. Reports comparing hydrophilic and hydrophobic acrylic IOLs have shown that the former has more severe PCO. On the other hand, we have previously demonstrated that the adhesion of LECs is favored on hydrophobic compared to hydrophilic materials. By combining these two facts and contemporary knowledge in PCO development via the EMT pathway, we propose a biomimetically inspired strategy to promote LEC adhesion without de-differentiation to reduce the risk of PCO development. By surface grafting of a cell adhesion molecule (RGD peptide) onto the conventional hydrophilic acrylic IOL material, the surface-functionalized IOL can be used to reconstitute a capsule-LEC-IOL sandwich structure, which has been considered to prevent PCO formation in literature. Our results show that the innovative biomaterial improves LEC adhesion, while also exhibiting similar optical (light transmittance, optical bench) and mechanical (haptic compression force, IOL injection force) properties compared to the starting material. In addition, compared to the hydrophobic IOL material, our bioactive biomaterial exhibits similar abilities in LEC adhesion, morphology maintenance, and EMT biomarker expression, which is the crucial pathway to induce PCO. The *in vitro* assays suggest that this biomaterial has the potential to reduce the risk factor of PCO development.

## Introduction

Cataract is the opacity of the crystalline lens or capsule of the eye, causing impairment of vision or even blindness. Cataract surgery, with damaged native lens extraction and IntraOcular Lens (IOL) implantation, is still the only currently available treatment. Nowadays, the conventional materials used for IOLs include PMMA (Poly(Methyl MethAcrylate)), silicone, hydrophobic acrylic, and hydrophilic acrylic polymers [1]–[5]. Secondary cataract, or Posterior Capsular Opacification (PCO), is the most common postoperative complication of cataract surgery. PCO involves the clouding of the posterior capsule by the lens epithelial cells (LECs), forming a thick layer on the IOL and causing loss of vision again. Although Nd:YAG laser capsulotomy has been used to treat PCO by creating a hole in the clouded lens capsule to allow light to pass to the retina. This method also potentially creates other complications such as damage to the IOL, higher intraocular pressure, cystoid macular edema, and retinal detachment [1], [6]. The problem of PCO has been a challenge to scientists and ophthalmologists for decades.

The biological basis of PCO has been investigated [7]. In the normal crystalline lens, the LECs attach to the anterior capsule and form a monolayer. The LECs are quiescent in a contact-inhibition status. During cataract surgery, the structure is broken and the residual LECs become active in proliferation and migrate into the space between the posterior capsule and the IOL. The LECs further undergo Epithelial-Mesenchymal Transition (EMT) and transdifferentiate to fibroblasts. These cells express α-smooth muscle actin and secrete collagen I, III, V, and VI, which are not normally present in the lens. The extracellular matrix network and the over-proliferated cells scatter light and lead to PCO. Another concept of tissue response to biomaterials has also been suggested to explain PCO formation [8]. Surgical trauma provokes the breakdown of blood–aqueous barrier (BAB) and the infiltration of macrophages and giant cells, further inducing foreign body reactions. These cells secrete cytokines including transforming growth factor β (TGF-β), and fibroblast growth factors (FGFs) which promote EMT and fibroblast transdifferentiation. At the final stage, the fibrous encapsulation of IOLs marks the end of tissue self-healing and the formation of PCO [7], [9].

PCO is known to be multifactorial. The incidence can be influenced by the patient's age or disease, surgical technique, and IOL design and material [10]. Research scientists and ophthalmologists worldwide have attempted to alleviate PCO development. These attempts can be categorized into the improvement of surgical techniques, the use of therapeutic agents, IOL materials and designs, and combination therapy [6].

The improvement in the surgical technique is mainly focused on the removal of LECs at the time of lens extraction. The proposed techniques, including aspirating/polishing anterior or posterior capsule, have been reported to delay but not to eliminate PCO for the reason that PCO is mainly caused by germinative LECs in the equatorial region rather than the displaced metaplastic LECs already on the posterior capsule [6]. Hydrodissection, injection of physiological saline fluid stream in-between the capsular bag and lens to facilitate the removal of retained cortical material and LECs, was shown to be important for PCO prevention [11]. However, it does not completely eliminate LECs.

The research of therapeutic agents is mainly focused on selectively destroying residual LECs without causing toxic effects to other intraocular tissues. The routes of administration can be direct injection into the anterior chamber, addition to the irrigating solution, impregnation of the IOL, or iontophoresis [12]. Unfortunately, a wide range of pharmacological agents as well as cytotoxic and therapeutic agents have shown the potential to prevent PCO *in vitro*, but exhibit toxic effects to the nearby ocular tissues *in vivo* [6]. The lack of selectivity currently limits their clinical use.

Scientists are also devoted to reducing PCO by developing IOL materials and designs. PCO was regarded as an inevitable consequence of lens implant surgery until 1993, when the clinical trial of the hydrophobic acrylic IOL (Acrysof IOL MA series, Alcon Laboratories) was conducted [10]. In 2000, Nishi and his colleagues proposed a method of preventing PCO development by square-edge IOL design, which involved the sharp-edge of IOL inhibiting cell migration to the optic part along the lens capsule [13]. The square-edge IOL was later improved with 360° design to prevent cell migration via haptic-optic junction and achieved a significant decrease in PCO formation [14]–[16]. However, more and more evidences are showing that the square-edge design can only delay rather than prevent PCO formation [17]–[19]. Recently, adhesion-preventing ring designs have been proposed to inhibit PCO by separating the anterior and posterior capsular flaps which allow aqueous humor to circulate in and out of the capsular bag and lead to LEC proliferation inhibition [20], [21]. However, the biological mechanism of these designs in PCO inhibition is yet to be investigated [22]. Nowadays, it is generally accepted that the square-edge design and hydrophobic acrylic material are better choices to inhibit PCO. PCO is, however, still a challenge to scientists and ophthalmologists.

The materials for IOLs require excellent optical properties for light transmission, mechanical properties for folding injection during surgery, and biological properties for preventing unfavored body reaction. Biocompatibility is generally accepted as the ability of biomaterials or medical devices to perform specific functions with appropriate host response [9]. For IOLs, the biocompatibility can be assessed in terms of uveal and capsular compatibility, which are the inflammatory foreign-body reaction of the eye against the implant and the relationship of the IOL with remaining LECs within the capsular bag, respectively [23]. Among the acrylic materials, the hydrophilic ones are superior to the hydrophobic ones in the uveal aspect, which is inversely related to inflammation [24]. However, the hydrophilic acrylic material is considered less capsular biocompatible, which is related to the higher incidence of PCO [25].

The molecular basis of high PCO incidences in hydrophilic IOL materials has been speculated by protein adsorption behaviors [26], [27]. Linnola *et al*. have shown that more fibronectin was adsorbed on the hydrophobic IOLs than on the hydrophilic ones [28]–[30]. Therefore, the hydrophobic IOL can be considered as bio-sticky (i.e. stick to the capsular bag via the adsorbed proteins or via the adsorbed adhesion protein-induced cell layer mechanism) [31]. This glue effect could possibly inhibit the migration of residual LECs tending to invade the posterior capsule from the haptic–optic junction [19], [32]. On the other hand, in the *in vitro* culture experiments, LEC differentiation is drastically accelerated if the cells are not well attached [33]. In this context, Linnola also proposed a “Sandwich Theory” model to control PCO [34]. In this model, a sandwich-like structure of fixed LECs between the lens capsular bag and IOL could be formed by selecting a sticky IOL material. The LECs regained the mitotically quiescent status and diminished eventually without provoking PCO.

The hydrophilic acrylic polymer, mainly composed by pHEMA (Poly(2-HydroxyEthyl MethAcrylate)), has several superior characteristics. Surgeons benefit from its foldability and controlled unfolding behavior. Patients suffer less from glistening and the glare phenomenon [1]. For the manufacturers, the rigidity in the dry state is helpful for easy machining. However, IOLs made from this material are prone to induce secondary cataract [32]. Therefore, it will be beneficial to improve the capsular biocompatibility of hydrophilic acrylic materials. With the hypothesis of “Sandwich Theory”, we assume that the ability to attract LEC adhesion is one of the key factors in PCO development.

Our strategy of PCO control is to improve the hydrophilic IOL capsular biocompatibility by creating a higher LEC affinity surface. We recently highlighted their particular properties in terms of adhesion forces, LECs adhesion, and tissue response as indicators of PCO development risk [27]. In this study, we propose a strategy to reduce the risk factor of PCO by functionalizing the surface of hydrophilic acrylic reticulated polymer of 25% water content (HA25) with a cell adhesion peptide containing the Arg-Gly-Asp sequence (RGD peptides [35]), and we characterized these surfaces using X-ray photoelectron spectroscopy and contact angle measurements. To evaluate the PCO control performance, *in vitro* LEC culture was performed and compared to a hydrophobic acrylic material (Glistening-Free polymer, GF) considered here as a negative PCO control. Additionally, in order to ensure that the surface functionalization process does not compromise the material properties, optical and mechanical properties of functionalized polymers were conducted and compared with control materials.

## Materials and Methods

**Figure 1:**
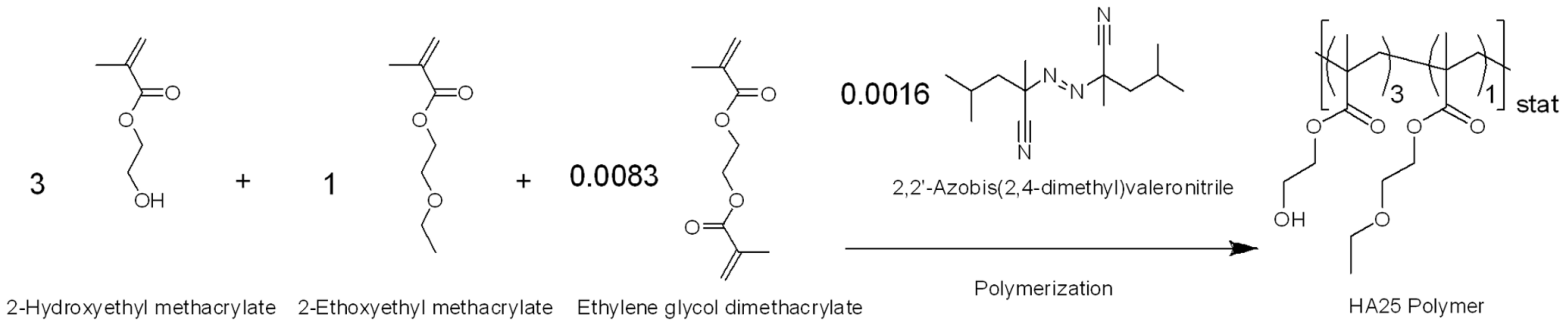
Fabrication and chemical structure of virgin HA25 material.

**Figure 2:**
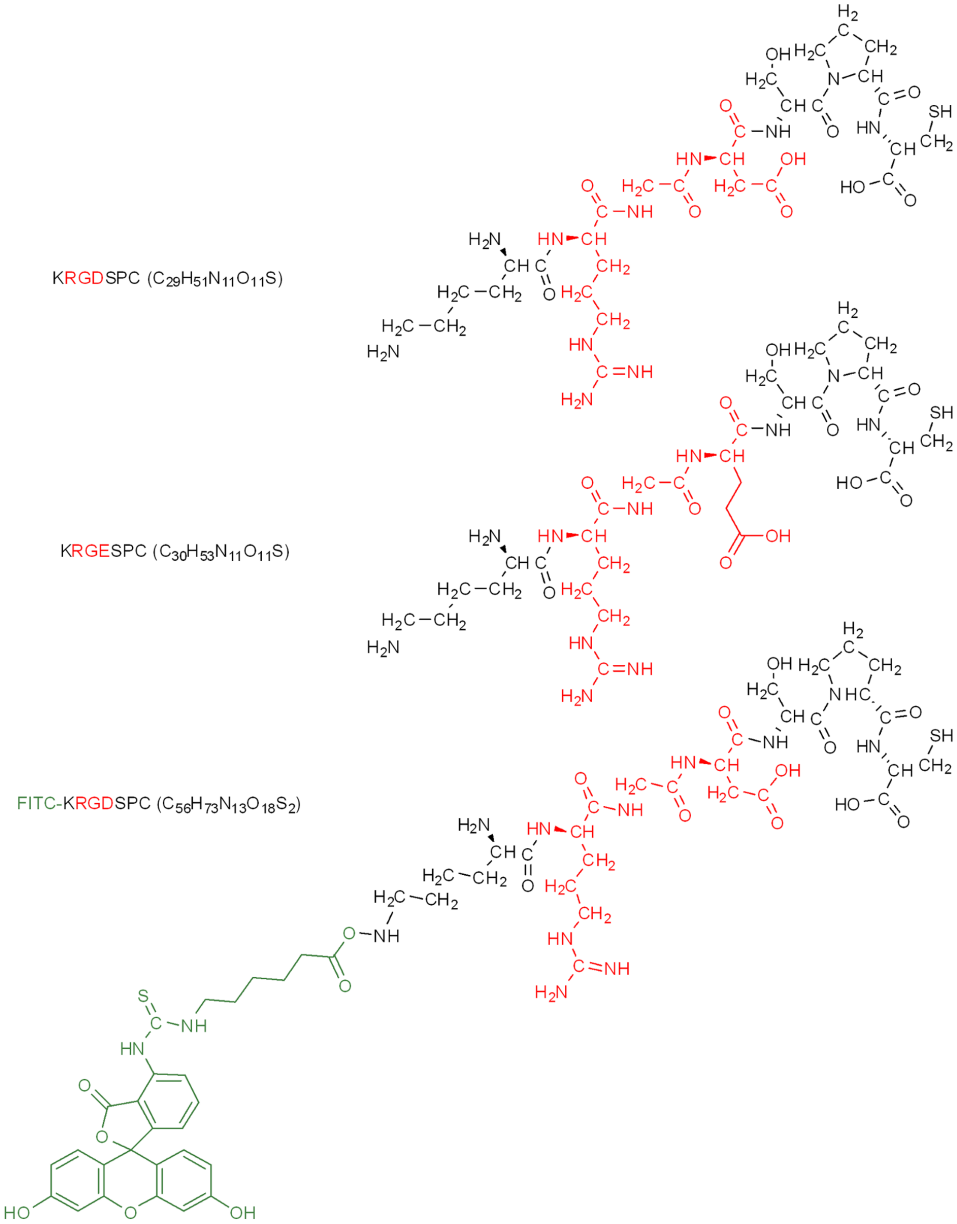
Chemical structure of RGD, RGE, FITC-RGD.

Cross-linked hydrophilic acrylic polymer of water uptake 25% (HA25) was obtained from Benz Research and Development (BRD, Sarasota, USA), and the cross-linked hydrophobic acrylic polymer of Glistening-Free (WO 2006/063994) was obtained from PhysIOL (Liège Science Park, Liège, Belgium). Disks were diamond-turned and milled down to a thickness of 1 mm and a diameter of 14.5 mm and were then treated according to procedures typically applied for IOL manufacturing. The HA25-based IOLs were obtained from PhysIOL. The chemical structure of virgin HA25 is shown in Fig. 1. KRGDSPC peptide (denoted as RGD), its negative control KRGESPC peptide (an analog of the RGD peptide without integrin-interacting function, denoted as RGE), and fluorescein-labeled KRGDSPC peptide (denoted as FITC-RGD) were purchased from Genecust (Luxembourg). The chemical structure of the peptides are shown in Fig. 2. Primary antibodies including mouse anti-α-SMA (ab7817), rat anti-tubulin (ab6160), and mouse anti-cytokeratin (ab668) were purchased from Abcam (Cambridge, UK). Fluorescent secondary antibodies Alexa Fluor 594 goat anti-mouse (A-11032) and Alexa Fluor 488 chicken anti-rat (A-21470) were purchased from Life Technology (Gent, Belgium). Porcine TGF-β1 (101-B1-001) was purchased from R&D systems (Minneapolis, USA) and rapamycin was purchased from Apollo Scientific (UK). The peptide-functionalized surface and its controls are listed in Table 1.

**Table 1:** Nomenclature of samples used in this study.

Name	Oxygen Plasma treatment	Peptide Incubation	Description
TCPS	N/A	N	Tissue culture polystyrene, used as normal LEC morphology/proliferation control
TCPS/TGF-β	N/A	N	LEC cultured onto TCPS with additional TGF-β (20 ng/µL) in normal culture medium, used as EMT positive control
TCPS/Rapamycin	N/A	N	LEC cultured onto TCPS with additional rapamycin (20 nM) in normal culture medium, used as EMT negative control
GF	N	N	Hydrophobic acrylic polymer Glistening-Free, used as PCO negative control
HA25	N	N	Hydrophilic acrylic polymer water-uptake 25%, used as PCO positive control
HA25 plasma	Y	N	HA25 treated with oxygen plasma, used for plasma effect evaluation
HA25-RGD ads	N	Y (RGD)	RGD adsorbed onto HA25, used for adsorption effect evaluation
HA25-RGD graft	Y	Y (RGD)	RGD grafted onto HA25, used for LEC adhesion promotion
HA25-RGE ads	N	Y (RGE)	RGE adsorbed onto HA25, used for adsorption effect evaluation
HA25-RGE graft	Y	Y (RGE)	RGE grafted onto HA25, used for a negative control of LEC adhesion promotion
HA25-FITC RGD ads	N	Y (FITC-RGD)	FITC-RGD adsorbed onto HA25, used for adsorption effect evaluation by fluorescence tracing
HA25-FITC RGD graft	Y	Y (FITC-RGD)	FITC-RGD grafted onto HA25, used for LEC adhesion promotion and fluorescence tracing

### Peptide grafting

**Figure 3:**
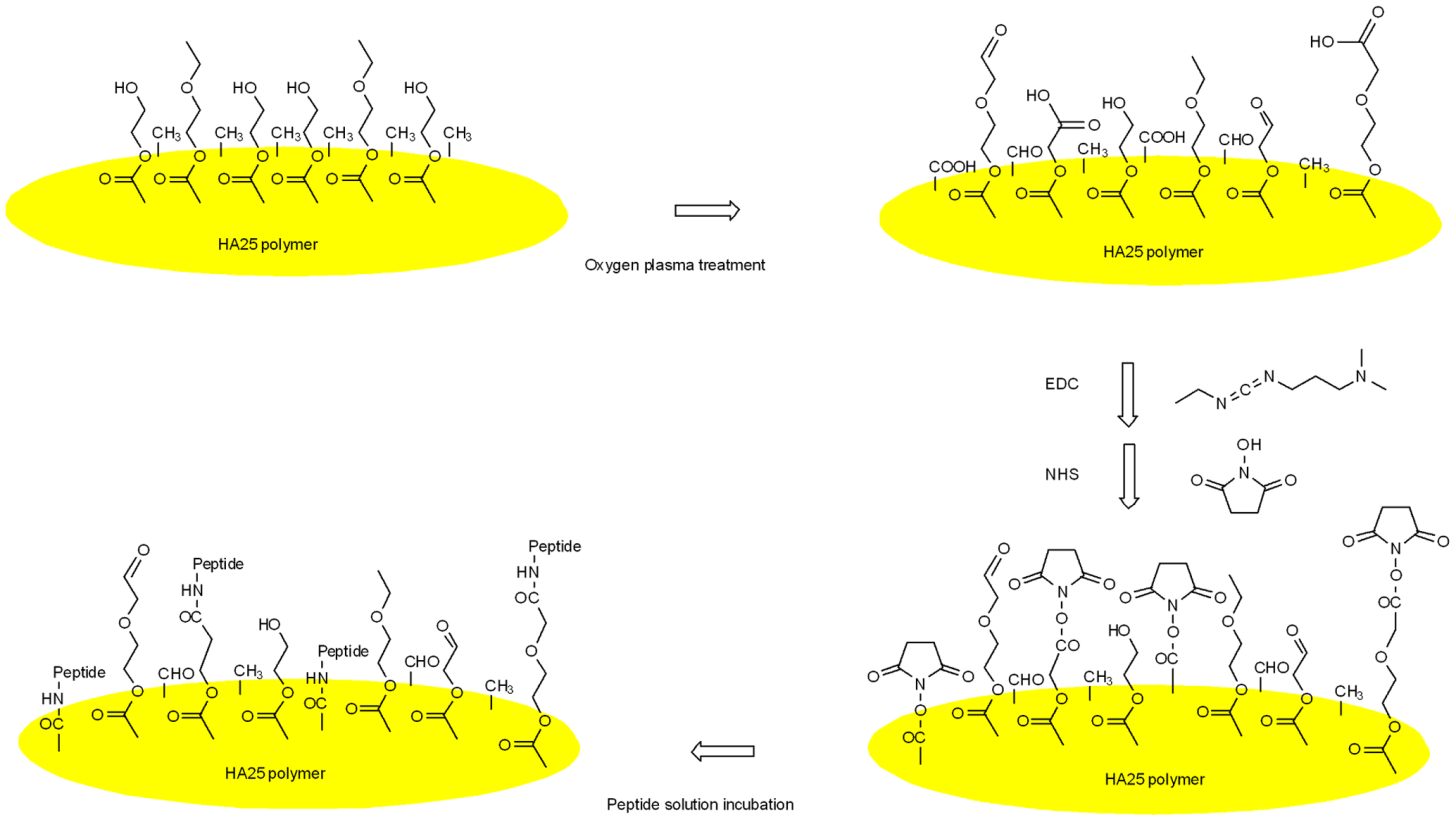
Chemical structure and reaction to prepare RGD surface-functionalized HA25 disk/IOL.

The schematic illustration of the peptide grafting procedure is shown in Fig. 3. IOL samples made by HA25 were specifically used in optical (optical bench measurement) and mechanical (haptic compression force, IOL injection force) properties analysis. Disk samples made by HA25 were used as models for all the other tests. The disk and IOL samples were rinsed with deionized water before overnight air drying. Dried samples were then placed into the chamber of radio frequency glow discharge (RFGD) instrument (customized, Europlasma). Surface activation by plasma treatment was driven at 200 W for 10 minutes. The flow rate of oxygen gas was set at 15 Sccm, and the system pressure was maintained at 50 mTorr. After plasma treatment, the samples were immersed into the coupling solution containing 1-Ethyl-3-(3-dimethylaminopropyl)carbodiimide (EDC) 150 mM, N-hydroxysuccinimide (NHS) 100 mM, and 2-(N-morpholino)ethanesulfonate (MES) 100 mM at 4°C overnight. After rinsing with MilliQ water, the samples were conjugated with peptides by incubation with 1 mM peptide solution for 24 hours at room temperature.

To remove the physically adsorbed peptides, the samples were extracted in deionized water combined with ultrasonication (ultrasonic cleaning) and elevated temperature (autoclave). The samples after peptide solution incubation were placed in a MilliQ water-filled chamber of ultrasonication (35 kHz, 60 W, Elmasonic One, Germany) for 1 hour. Then each sample was placed in a glass container filled with MilliQ water and subjected to autoclave (120°C, 1 bar) for 45 minutes. The autoclave treatment was performed for 10 cycles with fresh MilliQ water every time.

For the control samples (S1 Figure), peptide adsorption effect controls (RGD ads, FITC-RGD ads, and RGE ads) were made by direct incubation of virgin dried HA25 material with the same peptide solution followed by the same washing steps as mentioned above. Plasma effect control (HA25 plasma) was prepared only by the plasma and immersed in MilliQ water immediately after the treatment.

### X-ray photoelectron spectroscopy (XPS) characterization

A ThermoFisher Scientific K-ALPHA spectrometer was used for disk surface analysis with a monochromatized AlKα source (hν = 1486.6 eV) and a 200 micron spot size. A pressure of 10^−7^ Pa was maintained in the chamber during analysis. The full spectra (0–1150eV) were obtained at a constant pass energy of 200eV and high resolution spectra at a constant pass energy of 40 eV. Charge neutralization was required for all insulating samples. High resolution spectra were fitted and quantified using the AVANTAGE software provided by ThermoFisher Scientific.

### Cell culture

Lens epithelial cells (LECs) were isolated from the lens crystalline anterior capsular bag of pig eyes (Pietrain-Landrace pig; from Detry SA. Aubel, Belgium) as previously described [36]. The complete culture medium was composed of 85% Dulbecco's Modified Eagle's Medium (BE12-733, Lonza), 10% fetal bovine serum (10270-106, Gibco), 1% penicillin/streptomycin antibiotics (BE17-602, Lonza), 1% non-essential amino acids (NEAA) (BE13-114, Lonza), 1% sodium pyruvate (BE 13-115, Lonza), 1% Glutamax (35050 Gibco, Invitrogen, Oregon, United States), and 1% HEPES (17-737, Lonza, Vervier, Belgium). The cells were cultured in an incubator under the condition of 5% CO_2_ enriched atmosphere at 37°C. Trypsin-EDTA (Gibco, Invitrogen) was used for cell detachment after one rinse with PBS without calcium and magnesium (BE17-516, Lonza).

### Cell adhesion assay

The cell adhesion assay on polymer disks was previously described elsewhere [27]. The peptide-immobilized (i.e. grafted and adsorbed) polymer disk samples were cut into 14 mm diameter disks, washed and sterilized in PBS (with Ca^2+^ and Mg^2+^) (BE17-513, Lonza) at 1 bar, 120°C, for 21 minutes. The polytetrafluoroethylene (PFTE) cell culture (SIRRIS customized, Liège, Belgium) inserts with an inner diameter of 12 mm were also sterilized by autoclave. Each disk was put into a well of a 12-well culture plate (from Greiner Bio-One, Frickenhausen, Germany) and fixed by an insert for cell seeding. The LEC concentration was adjusted to 1.59×10^5^ cells/mL. For each well, 750 µL of cell suspension was added (1.4×10^5^ cells/cm^2^). The cells were seeded on surfaces without serum for 6 hours to allow the RGD peptides to act on receptors without the hassle of serum proteins. The unattached cells and the serum-free culture medium were removed after 6 hours of serum-free incubation. The remaining attached cells were further cultured in fresh complete medium for 3 days to allow cell spreading and proliferation. In order to evaluate the LEC adhesion, the cell culture was stopped for further immunofluorescence staining. The culture medium was removed, and the samples were carefully washed with PBS (with Ca^2+^ and Mg^2+^) in order to eliminate the non-adhering and dead cells.

EMT can be indicated by cell shape [37] and spatial distribution [38]. Epithelial cells are rounded/polygonal in shape and organized in clusters whereas mesenchymal cells are elongated/flattened in shape and scattered. However, if the cultured cells are close to confluence, the cells can adapt a distinct growth form and acquire overlapping cytoplasmic expansions. Therefore, for the EMT induction or inhibition by porcine TGF-β or rapamycin, the cell preparation and complete DMEM medium were the same as mentioned above. The conditions of porcine TGF-β 20 ng/µL and rapamycin 20 nM were tested by addition of each component into the TCPS culture flask. Moreover, in order to better observe the shape and spatial distribution of individual cells, the cells were cultured for 1 day rather than 3 days to avoid confluence.

### Immunofluorescence of EMT markers

The cell fixation step was performed with 4% paraformaldehyde in PBS at room temperature for 20 minutes. Cell permeabilization was performed with 0.5% Triton X-100 in PBS at 4°C for 20 minutes. Blocking was performed with 1% BSA in PBS at 37°C for 1 hour. Primary antibody incubation was performed in 0.05% Tween-20 in PBS solution with corresponding dilutions (1∶100 for αSMA, 1∶1000 for tubulin, and 1∶200 for cytokeratin) at 37°C for 1 hour. Secondary antibody incubation was performed in 0.05% Tween-20 in PBS solution with a dilution of 1∶200 at 37°C for 1 hour. The stained sample surface was observed with an IX81 optical inverted microscope equipped with a UPlanFL objective at x10 magnification with an XCite-iris IX fluorescence unit and a C-BUN-F-XC50 charge-coupled-device camera (Olympus Optical Co., Ltd). The size of each image was 625 µm×930 µm. The full image area corresponded to 581,250 µm^2^ (10,036,224 pixels) and was related to a cell coverage of 100%. At least three images per condition were acquired. For quantification of attached LECs on the surfaces, we used the image analysis software “CellSens” (Olympus). Threshold values were determined empirically by selecting a setting, which appeared similar to the original photomicrograph but with minimal background. After threshold selection, the resulting image was then converted to a binary image and the coverage % was reported by the software automatically. The colored merged images were generated by the channels combining function of the same software.

### Optical properties

The light transmittance test on disks was accomplished by keeping the samples hydrated and placing them onto a plastic 96-well plate for optical density scanning. Spectrum scan was set from 200 nm to 999 nm with 1 nm interval (PowerWave, BioTek). The absorbance was obtained and transformed to light transmittance after blank subtraction. The spectra were recorded from 370 nm to 999 nm.

The optical bench measurement protocol was described previously [39] and aims at verifying the preservation of the optical performance of the IOL after modification. The protocol consists of conditioning the neat and modified IOLs in physiological solution (0.9% NaCl, Baxter) for at least 24 hours, and analyzing with an optic bench (NIMO TR0815, Lambda X) their optical properties (optical power and contrast sensitivity, expressed by the modulation transfer function (MTF)). This test was performed according to ISO 11979-2. The IOLs (1 per IOL model) were positioned in a quartz cuvette filled with physiological solution (0.9% NaCl, Baxter). The measurement was performed at a 3.0 mm aperture and a spatial frequency of 100 cycles/mm.

### Mechanical properties

The mechanical tests on IOLs comprised evaluation of the lens foldability upon injection and the capacity of the lens haptics to support lens stabilization at different capsular bag sizes. They were designed and performed according to ISO 11979-3 and the detailed protocols were described previously [39].

Briefly, the IOL injectability was tested with the injection system Accuject 2.2 -1P (Medicel AG) at 21°C with a compression/traction mechanical bench (FL Plus Lloyd Instruments, Ametek) with a possible value variation of less than 0.05%, simulating surgical manipulation. The test equipment was supplied with a load cell of 100 N and operated with Nexygen FM software (Chatillon, Ametek, Inc.). The force applied by the haptics to the capsular bag (for simulated capsular bag size of 11.0 mm, 10.5 mm, 10.0 mm, and 9.5 mm) was estimated by a compression force tester (MFC-1385-IOL, Applied Micro Circuits Corp.). The possible value variation is smaller than 0.2%.

### Contact angle measurement

Before the measurement, the sample disks were rinsed in MilliQ water and then dried in an oven at 35°C for 2 days. The aqueous contact angle was measured (4 measurements/water droplet, 2 droplets/disk, 3 disks/sample) with a dual-gradient density contact angle meter (DGD Fast/60) coupled to WindropCC software (Digidrop, GBX). The static contact angles were measured by the water-droplet method after deposition of 15 µL deionized water on the dry disk surfaces.

### L929 cell viability test with conditioned media (MTS Assay)

Each conditioned medium was prepared by immersing a 14.5 mm polymer disk into 1.2 mL of complete culture medium (87% Dulbecco's Modified Eagle's Medium (BE12-733, Lonza), 10% fetal bovine serum (10270-106, Gibco), 1% penicillin/streptomycin antibiotics (BE17-602, Lonza), 1% sodium pyruvate (BE13-115, Lonza), and 1% Glutamax (35050, Gibco)) in a 12-well culture plate and incubated at 37°C, 5% CO_2_ for 3 days. These conditioned media were added to wells containing adherent mouse L929 cells. Mouse L929 cells were precultured in a 96-well culture plate. The seeding amount for each well was 2000 cells in 100 µL of culture medium. After one day in culture, the medium was removed and the wells were replenished with 100 µL of disk-conditioned medium or unconditioned fresh medium as controls (100% viability). The cells were cultured for another 3 days. The medium was then replaced by fresh DMEM/F-12 (21041-025, Gibco) and an additional 20 µL of MTS (3-(4,5-dimethylthiazol-2-yl)-5-(3-carboxymethoxyphenyl)-2-(4-sulfophenyl)-2H-tetrazolium) solution (G5421, Promega) was added. The MTS compound was bio-reduced by cells into a colored formazan product that is soluble in culture medium. The quantity of formazan product is related to viable cell population. The cells were incubated in a CO_2_ supplemented incubator for 1 hour and absorbance was read with a microplate reader (PowerWave, BioTek). The 490 nm absorbance was obtained and the cytotoxicity was calculated and normalized from the absorbance of control samples taken as 100% (cells in identical culture environment but with unconditioned medium).

### Statistical Analysis

For all experiments, at least 3 disks/IOLs replicate were prepared and analyzed independently. The quantified data were subjected to statistical analysis with Prism software (GraphPad, San Diego, USA). Unpaired t-test was applied to compare between test groups using a 95% confidence interval and two-tailed P value. Not significant (P>0.05) is denoted as “ns” and P values smaller than 0.01 and 0.001 are denoted as 2 and 3 stars, respectively. One-way ANOVA was applied to compare among test groups using a 95% confidence interval and Tukey post-test. The error bar of all the graphs presented stands for standard deviation.

## Results

### Characterization of peptide immobilization by XPS

**Figure 4:**
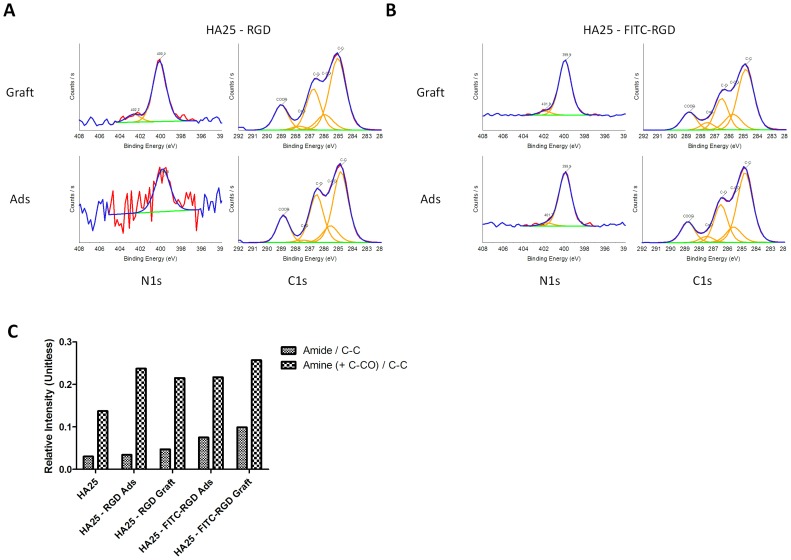
XPS analysis of peptides immobilized onto HA25. (A) XPS C1s (left) and N1s (right) spectra of the HA25 - RGD grafted (upper) and adsorbed (lower) sample. (B) XPS C1s (left) and N1s (right) spectra of the HA25 - FITC-RGD grafted (upper) and adsorbed (lower) sample. (C) From C1s spectra in (A) and (B), quantification of amide/C-C and (amine + C-CO)/C-C ratios for each material.

XPS was employed to determine the surface chemical composition of HA25 before and after RGD peptide immobilization. The XPS spectra of the different statuses of chemical modifications were collected and their element compositions were calculated and shown in Fig. 4 and Table 2. The peak deconvolution results of the C1s spectra and the N1s spectra are shown in Fig. 4A.

**Table 2:** Experimental atomic composition obtained by XPS analysis.

Atomic %	C%	O%	N%	Si%
HA25	70.8	25.2	0.4	3.6
HA25 - RGD ads	70.7	25.9	0.2	3.2
HA25 - RGD graft	70.0	26.2	1.0	2.8
HA25 - FITC-RGD ads	69.1	24.3	2.4	3.7
HA25 - FITC-RGD graft	70.0	23.5	3.1	3.0

### LEC adhesion assay

**Figure 5:**
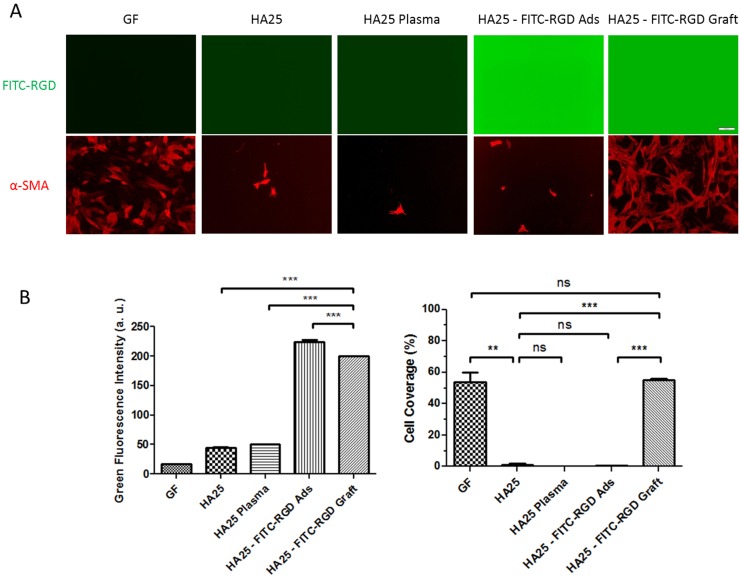
LEC adhesion assays of the polymer disks immobilized with FITC-RGD. (A) Fluorescence microscopy images in green channel (detecting FITC-RGD) and red channel (detecting α-SMA) (bar  = 100 µm). (B) Quantification of the cell coverage ratio (red channel) and the average fluorescence intensity (green channel) from three independent images shown in (A).

**Figure 6:**
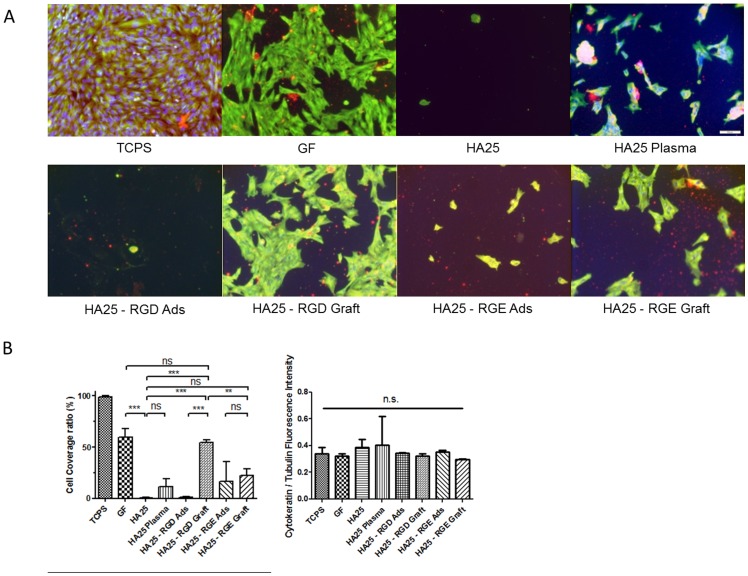
LEC adhesion assay of the polymer disks immobilized with RGD or RGE. (A) The fluorescence images of LEC-adhered surface (cytokeratin in red, tubulin in green, nucleus in blue) (bar  = 100 µm). (B) Quantification of the cell coverage ratio (green channel) (left) and the normalized EMT marker expression profile (red channel/green channel) (right) from three independent images shown in (A).

**Figure 7:**
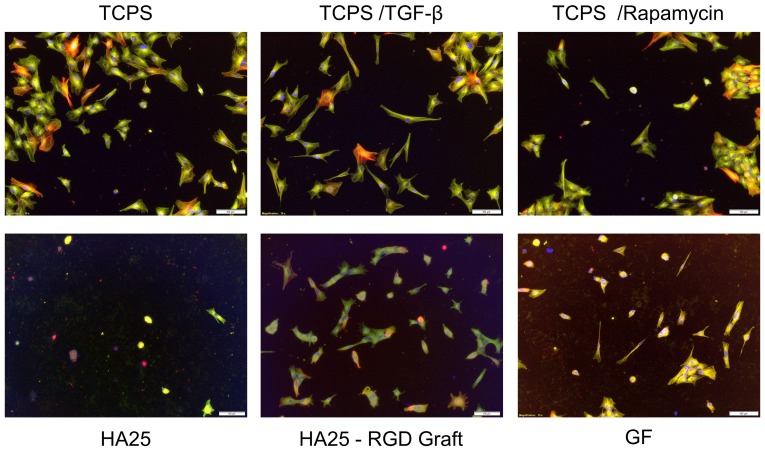
LECs adhesion on RGD grafted disk in contrast with EMT inhibitor (Rapamycin) or promoter (TGF-β) treated cells on TCPS. (α-SMA stained in red, tubulin in green, nucleus in blue) (bar  = 100 µm).

The bio-adhesive function of the RGD peptide was evaluated by culturing LECs onto the surface-functionalized polymer disks. After fixation and immunofluorescence staining, images were taken and the cell coverage ratios were calculated (Figs. 5, 6, and 7). The TCPS stands for Tissue Culture PolyStyrene surface optimized for cell culture and serves as a reference for LEC proliferation and morphology. As expected, the cells adhered least onto the virgin HA25 surface (HA25). As long as RGD peptides were grafted (HA25 - RGD Graft), the cell adhesion increased significantly. Moreover, the proliferated LECs clustered and spread in a similar fashion on the hydrophobic GF control (Glistening-Free (GF) polymer), providing evidence that the surface modification with RGD peptide facilitated the morphology maintenance as well as the adhesion of the porcine LECs. From this assay, we found that the RGD-grafted sample exhibited higher LEC adhesion than the adsorbed sample did (HA25 - RGD Ads) and its coverage ratio was similar to that of the hydrophobic material GF (Fig. 5). In addition, enhanced LEC adhesion was promoted specifically by RGD peptide grafting and the expression of epithelial biomarker cytokeratin remained unaltered (Fig. 6). On the other hand, the RGD grafted sample exhibited better LEC morphology maintenance than the starting material HA25 and showed similar spatial distribution and shape to the hydrophobic material GF (Table 1 and Fig. 7).

### Optical properties

**Figure 8:**
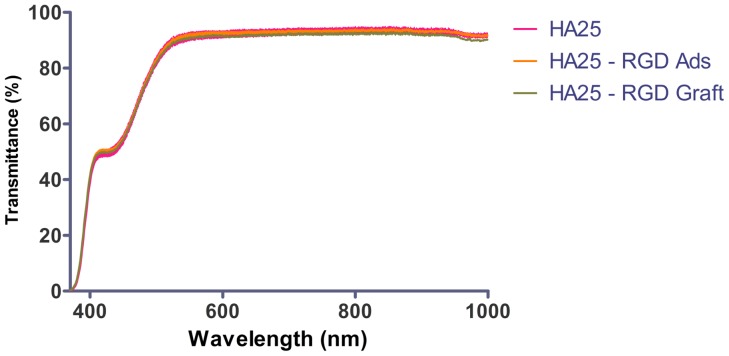
Light transmittance spectra of the neat and modified disks (No significant difference among 3 groups by ANOVA).

The light transmittances of the disks with different peptide surface immobilized statuses were measured (Fig. 8). The disks were homogenously transparent with no color derivation, and presented a light transmittance of greater than 80% from green light to red light spectrum. The RGD-immobilized samples (HA25 – RGD Ads and HA25 – RGD Graft) exhibited the same optical properties as the virgin polymer did (HA25). The small thickness variance in the polymer disks may cause a homogenous effect in the entire transmittance spectrum. The decrease in light transmittance in blue light was a result of the “Blue Filtering” design of the bulk polymer disk.

In addition, from the optical bench measurement on IOLs, both RGD grafted and adsorbed samples exhibited no impact on the optical power, expressed in diopters, and the contrast sensitivity, expressed by the MTF, of the lenses (Table 3). These parameters remained within the pre-established industrial tolerances after IOL modification.

**Table 3:** Neat and modified IOLs analyzed with an optical bench.

Sample name	MTF	Theoretical diopter, D	Measured diopter, D
**HA25 n° 1**	0.64	20,50	20.35
**HA25 n° 2**	0.65	20,50	20.78
**HA25 n° 3**	0.65	21,00	21.08
**HA25 - RGD ads n°1**	0.60	22,50	22.71
**HA25 - RGD ads n°2**	0.65	22,50	22.23
**HA25 - RGD ads n°3**	0.63	18,50	18.38
**HA25 - RGD graft n°1**	0.65	22,50	22.62
**HA25 - RGD graft n°2**	0.61	15,50	15.57
**HA25 - RGD graft n°3**	0.65	21,00	21.10

Acceptable tolerances: MTF >0,43; diopter theoretical ±0,34D.

### Mechanical properties

The IOL injection force is the force applied to inject the IOL into the capsular bag from the injector. This force may change with a different injector, IOL geometrical design, buffer system, material water uptake nature, and surface friction. The higher the injection force, the more risk of damage in the IOL (scratches, haptic, or optic cracks) or in the injection system (cartridge break). No damage was observed in both the optic and haptic part of the IOL and all forces are close to 14 N, which was within the applied industrial criteria for this material and lens model. The test result showed that RGD immobilized-IOLs exhibited flexibility compared with the initial lens.

**Figure 9:**
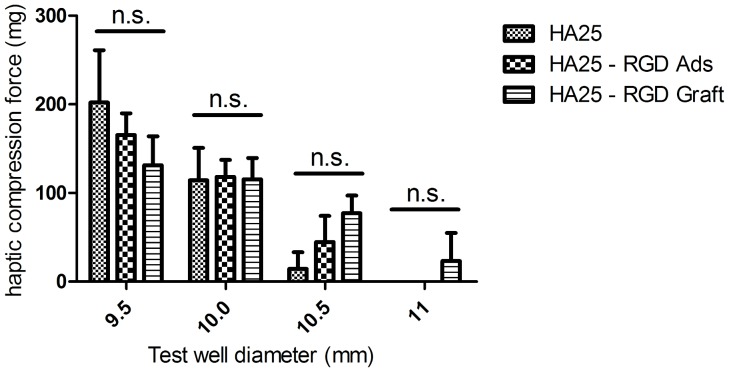
Haptic compression forces of IOLs collected for various simulated capsular bags sizes.

The haptics are designed to fix the IOL inside the capsular bag and the haptic compression force is the force that haptics applies to the capsular bag. Given the intrinsic deformability of hydrophobic and hydrophilic acrylic IOL materials, lens haptics is compressed in order to compensate capsular bag variations. The test result showed that RGD immobilized IOLs exhibited similar compression forces to different test wells compared to the initial IOL (statically non-significant by 1-way ANOVA test) (Fig. 9).

### Contact angle measurement

**Figure 10:**
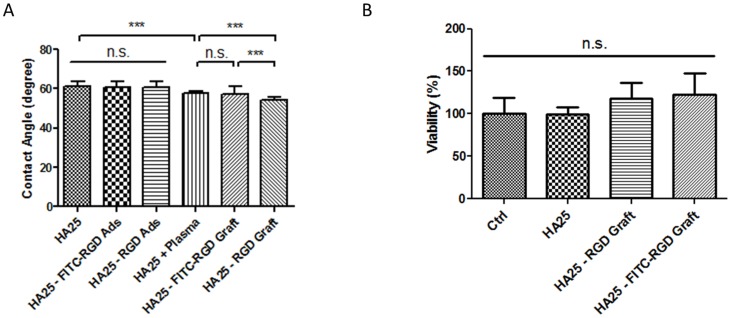
The water contact angle (A) and the cytotoxicity potential (B) of the neat and modified disks.

To verify whether the surface functionalization altered the hydrophilicity of the polymer, the water contact angles at the surface of the disks were measured after RGD peptide grafting. Samples of different RGD-functionalized surface were tested by the water-droplet method (Fig. 10A). The surfaces were hydrophilic and presented contact angles between 61° to 54°. The difference between the contact angles of peptide-adsorbed samples and the untreated HA25 disk was not statically significant using ANOVA test, indicating that the surface hydrophilicity was approximately the same. The plasma treated surfaces, however, became more hydrophilic. In addition, the RGD peptide grafted surface became even more hydrophilic than the virgin or plasma treated surface.

### MTS cytotoxicity assay

The evaluation of the cytotoxicity of disk-conditioned media on L929 cells was adapted from ISO 10993-5 (2009). From Fig. 10B, the viability percentages of all groups were above 70%, suggesting that the conditioned media were not cytotoxic. The surface-functionalized samples created by peptide immobilization did not release active substance(s) into the conditioned media.

## Discussion

### Strategic selection of bio-adhesive IOL to control PCO

Surface modification of IOLs to prevent PCO is considered as a simple and safe method because it requires no manipulation within the eye and no application of harmful agents during IOL implantation [6]. Bio-passive components such as COO^−^/SO_3_^−^ functional groups [40] , titanium [41], heparin [42], PFTE/fluorocarbon [43], poly(ethylene-glycol) and its derivatives [36], [44], [45] have been suggested to be generated, grafted, or coated onto the IOL materials to reduce cell adhesion or inflammatory reactions [2]. Recently, bio-active substances including anti-TGFβ2 antibody [46], [47], sulfadiazine (as a mimic of matrix metalloproteinases) [48], and 5-fluorouracil (an antimetabolite drug) [49] have been proposed to be immobilized onto IOL surfaces to block the processes involved in PCO. Despite applied similar surface functionalization technologies, we propose another strategy: the use of RGD peptides to create a bio-adhesive IOL material to aid the tissue repair and regenerate the native monolayer structure of LEC.

The RGD peptide has been suggested to specifically and safely promote cell adhesion. First, the RGD sequence is the functional motif of fibronectin [28]–[30], which is found to be adsorbed abundantly onto hydrophobic IOLs. Surface functionalization by RGD peptides is a biomimetic strategy to restore the LEC monolayer structure. In addition, the RGD modification method has been widely proposed in orthopedics, cancer diagnostic and therapy research fields to favor cell adhesion onto material surfaces [50]–[64]. Therefore, the fundamental safety profile of the peptide has been constructed from basic researches. Moreover, although the RGD-based drug Cilengitide failed in efficacy evaluation in Phase III [65], the safety tests of this peptide have already been passed in the clinical trial. From the current information, we speculate that the RGD peptide can promote LEC adhesion without toxic effects.

On the other hand, because RGD is recognized by numerous integrins in various cell types, different types of cells may non-discriminatorily attach to RGD-functionalized surfaces [66]. The lack of biological specificity renders the RGD-based strategies not fully optimized for controlling more integrated processes, for example cell differentiation [67]. To our knowledge, there is no simple peptide sequence that can specifically attract LECs. Therefore, it is possible that macrophages and fibroblastic cells, which migrate from uveal tissues into the capsular bag [68], could also attach to the RGD-grafted HA25 surface as BAB breaks down during the surgery. However, LECs could take advantage of binding to the RGD peptide grafted IOL temporally and spatially. These cells originated outside of the capsular bag are found to be maximized inside around 1 to 3 months postoperatively [7] whereas literature suggests that cell adhesion promoted by RGD becomes irreversible after 24 hours of incubation[69]. The pre-existing LECs, which are agitated during cataract surgery, would be the predominant cell type in the capsular bag, interacting with the grafted RGD peptide on HA25 polymer and occupying most of the available RGD sites on the surface, theoretically. Moreover, BAB alteration can be minimized by combining the clear corneal incision surgical method [70], which involves an incision in the plane of the cornea without altering uveal tissues [71]. Therefore, the risk of adhesion of non-epithelial cell types on RGD functionalized surface can be reduced. However, the deduction is based on the *in vitro* literature and needed to be verified by *in vivo* experimental data.

The surface functionalization process by plasma treatment and EDC/NHS coupling was chosen for its reproducibility and effectiveness. The advantages of applying plasma treatment in the surface modification are reliability, reproducibility, relative cost-effectiveness, and applicability to different sample geometries [72]. In the ophthalmological field, the plasma treatment has been shown to be able to graft specific molecules to prevent LEC adhesion [41], [44]. Nevertheless, the oxidation of acrylic IOLs could also control PCO in animal model [73]. In the present case, the combination of oxygen and plasma to perform surface functionalization has been proposed. Theoretically, oxygen plasma creates more oxygen-containing functional groups and transforms hydroxyl groups into carboxylic groups, which are ready for the peptide coupling reaction mediated by EDC and NHS. On the other hand, the EDC/NHS coupling method is widely applied in biological research and the detailed reaction mechanism is known. The conjugation can be made from the amide bond between the amine group of the RGD peptide and the carboxylic acid group of the oxygen plasma treated polymer [52], [56]–[61], [63], [64], [74], [75]. Therefore, the stepwise process was applied to our sample preparation method to graft RGD peptides onto the IOL material surface (Fig. 3).

There is another reason to adapt surface functionalization technology in PCO control. The surface modification strategy is superior to the pharmaceutical drug loading methods in terms of dose control [76]. For the patients applying higher diopter, the IOL is thicker and the volume to surface ratio is higher. Therefore, the drug releasing kinetics and the overall drug loading dose may vary case by case, and is hard to standardize in manufacturing by industry and regulation by government. Therefore, we are further convinced that surface functionalization with RGD peptides to create a bio-adhesive surface is a simple and safe way to control PCO.

### RGD peptide-grafted IOL material characterized by XPS

XPS analysis was performed to characterize the surface bound status (grafted or adsorbed) of the RGD peptides and to verify the effectiveness of the sample preparation procedure. Biomimetic grafting was performed by means of a three-step reaction procedure: creation of COOH functions onto the HA25 surface using plasma treatment, grafting of the coupling agent, and conjugation of the RGD peptides (Fig. 3). As shown in Fig. 3, this grafting method between the polymer surface and RGD or FITC-RGD resulted in an amide linkage.

The theoretical atomic compositions of RGD and FITC-RGD peptides were 56% C, 21% N, 21% O, 2% S and 64% C, 15% N, 19% O, 2% S, respectively (Fig. 2). On the other hand, RGD and FITC-RGD-grafted HA25 surfaces exhibited increased nitrogen element (Table 2) in comparison with virgin HA25 and RGD-adsorbed HA25.

As expected, the amount of nitrogen was higher in the case of FITC-RGD (grafted or adsorbed) than in the case of RGD (grafted or adsorbed). Each surface exhibited the expected elements with, additionally, a non-negligible pollution of Si, which had no impact on components including nitrogen. On the other hand, the virgin HA25 surface showed an N pollution (Table 2), which probably originated from additives (polymerization initiators or compounds used for blue filtering) [1], impurities, or contaminants upon storage and transportation [77].

The fitting components of C1s spectra and the N1s spectra are shown in Fig. 4A and 4B. The C1s components were assigned according to the literature: C-C, 284.9 eV; C-CO + amine, 285.8 eV; C-O, 286.6 eV, amide, 287.6 eV; COOR, 288.9 eV [78]–[80]. As for N1s, the assignment was amine, 400 eV; amide 402.5 eV [78], [81], [82].

The peak fitting components of the C1s spectra reflected the increased amide linkage between the peptides and the polymer (Figs. 3 and 4).

Moreover, the amide/amine increased in the case of HA25 - RGD graft (0.219) in comparison with HA25 - RGD ads (0.143) (Fig. 4C). The same statement is also applicable in the case of FITC-RGD peptide. The grafted sample exhibited a higher amide/amine ratio (0.385) than the adsorbed one (0.345), further illustrating that the conjugation occurred between the polymer and the targeting molecules (Fig. 4B, 4C).

The N1s fitting peak also showed a more defined peak in the case of HA25 - RGD graft (in comparison with HA25 - RGD ads) with one amide and one amine component (Fig. 4A). With FITC-RGD peptides grafted onto HA25, the amide component increased in comparison with HA25 - FITC-RGD ads (Fig. 4B). The N1s peak of HA25 FITC-RGD ads was well defined considering that the peptide had an additional nitrogen percentage.

For the RGD and FITC-RGD data set, although the existence of the amide peak (at 402 eV) cannot conclude conjugation, we still observed that the amide peak increased in the grafted sample (Fig. 4A, 4B), which is also complementary supporting evidence of conjugation as we have discussed in C1s fits. By combining C1s and N1s fitting results, we conclude that the RGD grafting process was effective to make the RGD surface grafted, rather than adsorbed, onto the HA25 polymer.

### LEC adhesion enhanced by grafting of peptides and potential of controlling PCO

Our proposal is to use the RGD peptide to create a bio-adhesive IOL material and to examine the LEC response to evaluate the PCO development risk indicators *in vitro*. Therefore, we used the commercially available hydrophobic IOL material (GF) as a low PCO level reference and the starting material, hydrophilic IOL HA25 (virgin polymer), as a high PCO level reference. In addition, in order to know the status of normal LECs *in vitro*, tissue culture grade polystyrene (TCPS) was also used for the maximum growth control.

On the other hand, the type of RGD peptide linkage onto the HA25 polymer is important. Non-covalent adsorption is sometimes appropriate for drug delivery applications [83]. However, in our case, covalent bond formation is more desirable for several advantages including higher stability [84], better cell adhesion promoting ability [85], and lower potential of uncontrolled desorption of RGD peptides in physiologic environments [86]. Therefore, the EDC/NHS coupling step was introduced to ensure the covalent bond formation between the RGD peptides and the HA25 polymer.

The bio-adhesive IOL material made by the grafting of RGD peptides onto the HA25 surface (HA25 – RGD Graft) promoted LEC adhesion comparable to the level achieved by the hydrophobic material (GF). The *in vitro* LEC adhesion assay of the FITC-RGD-immobilized surfaces (Fig. 5A) showed that the LEC adhesion was only promoted in FITC-RGD-grafted sample. The fluorescence backgrounds of the control samples without fluorescent peptide treatment (GF, HA25, and HA25 plasma) were significantly lower than the samples with fluorescent peptide treatment (FITC-RGD ads and FITC-RGD graft), suggesting that fluorescence is an appropriate tool to trace the peptides. The difference in fluorescence backgrounds between GF and HA25 may have been a result of the difference in their chemical compositions. The green fluorescence intensity of the sample reflected the amount of the FITC-RGD peptide present. In the case of the RGD-adsorbed sample, the high fluorescence may have resulted from the sorption phenomenon (i.e. molecules penetrated into the inner space of the hydrophilic acrylic polymer) [87]. Although the extreme condition of autoclaving had been applied to remove the surface-adsorbed peptides, a large amount of FITC-RGD peptides was “sorbed” inside the polymer, which could explain the low cell coverage of the FITC-RGD-adsorbed surface. In addition, RGD-adsorbed surfaces have been reported to have a lower ability to promote cell adhesion [85] because of the low stability, unstable links, and uncontrolled desorption of biomolecules in physiological environments.

By image quantification (Fig. 5B), the cell coverage percentage of the FITC-RGD-grafted sample (HA25 – RGD Graft) increased to the level observed on the hydrophobic material (GF), whereas the plasma treatment (HA25 Plasma) and the adsorbed (HA25 - RGD Ads) samples shared the same LEC adhesion level with the virgin polymer (HA25). The same observation was also made in the RGD-immobilized surfaces (Fig. 6A). The RGD sequence, rather than integrin non-interacting RGE, could promote LEC adhesion, which indicates the specificity of the surface functionalization. Therefore, we confirm that the LEC adhesion was promoted specifically from the grafting of the RGD sequence. The spreading of LECs could be seen only in the RGD-grafted sample and GF sample, illustrating the proper adhesion. In contrast, the LECs on the virgin polymer (HA25) or plasma-treated (HA25 Plasma) polymer showed a rounded shape, suggesting loose adhesion. In addition, the similarity of LEC coverage level between the RGD-grafted hydrophilic material (HA25 – RGD Graft) and the hydrophobic material (GF) (Fig. 6B) would imply similar capsule-IOL adhesive interaction mediated by LECs, and therefore similar low incidences of PCO.

The bio-adhesive RGD-grafted HA25 material shared similar EMT marker expression to the hydrophobic material level as well. After cataract surgery, the remaining LECs undergo EMT and express different proteins during PCO formation [88]. The progression of EMT can be detected with these protein biomarkers. For example, α-SMA is an acquired marker and cytokeratin is an attenuated marker during EMT [88]. The background expression of α-SMA is observed in porcine LECs as reported in other mammals [89]. Comparing with the hydrophobic sample or the TCPS sample, the LECs on the RGD-grafted hydrophilic sample do not acquire more α-SMA (Fig. 5A). On the other hand, the expression of cytokeratin was not attenuated in the RGD-grafted sample compared with the controls (Fig. 6B). Therefore, the biomarker assays of EMT illustrated no difference between the RGD-grafted hydrophilic material and the hydrophobic material, which would imply a low chance of undergoing EMT and induce PCO.

In addition, the epithelium morphology evidence showed that the bio-adhesive IOL material did not promote EMT. The native EMT status of LECs is cuboid-like with evident cell-cell adhesions [90]. Cultured *in vitro* or stimulated by TGF-β, the LECs disintegrate from the clustered structure and become fibroblastoid spindle-shaped cells. From the literature, the compound rapamycin is suggested to prevent PCO by inhibiting EMT [91], [92]. Therefore, the porcine TGF-β and rapamycin-treated LECs on the TCPS surface could be used in our experiment as the PCO-positive and PCO-negative controls, respectively (Fig. 7). Although the porcine LECs used in this assay were naturally spindle-shaped [93], the LECs on RGD-grafted surface were not further elongated compared to those on the TCPS sample. In addition, the LEC spatial distributions and the morphologies were similar between the RGD-grafted and GF samples, implying a low potential to undergo EMT and induce PCO.

The relationship between RGD peptides and EMT remains unclear from the literature. For the epithelium interacting with soluble RGD peptides in the perspective of EMT, contradictory results have been reported. One study suggested that EMT is favored by finding that TGF-β1 activated proteolysis of the L1 cell adhesion molecule (L1CAM) induced its RGD-motif binding to integrin and triggering the EMT pathways [94]. However, in another report, blockage of integrin by soluble RGD peptides inhibited the human hepatic epithelial carcinoma from acquiring a mesenchymal phenotype and protein marker vimentin, in a system of co-culture with mesenchymal stem cells [95]. In addition, studies on associating EMT with the integrin expression level of LEC are controversial [96], [97]. Furthermore, the soluble RGD peptides did not interfere with the avian LECs undergoing EMT in an ex vivo assay [97]. In contrary, the soluble RGD peptides blocked the TGF-β1-induced EMT in mouse mammary gland cells [98]. As for the grafted RGD peptides, there has been no precedent to predict the EMT effect. Therefore, direct assessment of EMT of the LEC response to grafted RGD peptides is needed, and our preliminary experimental results showed no significant changes in biomarker expression and morphology, at least under our experimental conditions.

### Immobilization of peptide not altering its functions required for IOL implantation

As a candidate of a new biomaterial in ophthalmic implant, the peptide-immobilized polymer should possess appropriate optical properties, mechanical properties, and biocompatibility. Since the starting material, HA25, is a conventional biomaterial used in IOLs, it is appropriate to use it as a control to investigate the impact of the peptide surface functionalization process.

Surface modification on IOLs by ion beam or plasma methods has been proposed to improve the surface hydrophilicity or biocompatibility [42], [99]. However, it is still risky to have color deviation after coating [41]. In our case, the grafting of RGD peptides onto the IOL surface did not change the light transmittance spectrum partially or globally (Fig. 8), illustrating no color deviation and high transparency as the starting material. The transmission spectrum in the 550 nm to 999 nm was greater than 90%, which is similar to the literature [46]. The transmittance drop in the range between 350 nm and 550 nm was due to the presence of “blue filtering” chromophore copolymerized within the IOL material aiming to prevent the retina from the toxic blue light. In addition, UV-light filter is also typically copolymerized to filter the light up to 350 nm. The present data demonstrated that the surface functionalization procedure of plasma treatment and RGD peptide grafting did not change the light transmittance of the bulk material.

An intraocular lens is intended to restore the vision of the patient, and its optical parameters such as diopter are calculated prior to implantation. Therefore, any kind of surface modification in the lens should not be detrimental for its optical performance. The optical power of an IOL is expressed in diopters and the industrial tolerances, inspired by the ISO 11979-2, should be respected. The data from the optical bench measurement demonstrated that the experimental optical power of the neat and modified lenses was preserved and remained within the tolerances (D_experimental_  =  D_theoretical_ ±0.34D), suggesting no IOL curvature or refractive power deviation as a result of the modification.

Indirectly, the preservation of the contrast sensitivity of the optic, expressed by the MTF, argues for good surface quality, i.e. homogeneous, low roughness, the latter parameters being most frequently associated with contrast sensitivity deviations [100].

Although plasma treatment and surface coating have been applied to improve surface function in biomaterial studies, there are still evidences showing that these modifications may alter the mechanical properties of the bulk media [101], [102]. As for the IOL study, this aspect becomes important because changes in mechanical properties may lead to implantation failure during (failure of injection system, unfolding) or after cataract surgery (dislocation of IOL, damage of IOL or lens capsule). Therefore, the mechanical properties tests were aimed at verifying whether the RGD-grafted IOL is still suitable for ophthalmological implantation. The measurement of IOL injection forces is a part of the standardized testing to ensure the safety of the device during the implantation. From our data, the injection forces of the test groups were close to 14 N, falling into the normal range of the hydrophilic IOL [39] .

The measurement of haptic compression is also a part of the standardized testing of IOL performance to simulate its behavior in *vivo*. The haptic compression force should neither be too high to damage the capsule bag nor too low to unfix the IOL. Reports have shown that the maximum force loading of lens capsule is between 400 to 800 mg, compared with 23 to 131 mg in our RGD-grafted IOL [103]. This low force range ensures the mechanical safety of the lens capsule. Additionally, the haptics should exert a steady force to different sizes of capsular bags for different patients. The forces of the RGD-immobilized IOLs exhibited stability as the virgin IOL, illustrating appropriate mechanical properties. On the other hand, striae formation (caused by high extension forces) in the posterior capsular after surgery may provide a chance for LEC migration and lead to PCO formation. The lower compression force may lead to lower circumferential pressure on the posterior capsule, which minimized the striae formation. The observed forces in our RGD-immobilized IOLs were close to those in the virgin IOL models.

Non-cytotoxicity is a general biocompatibility requirement for all medical implants. The MTS assay is widely applied to evaluate the cell viability by relating the intracellular dehydrogenase activity to the living cell population [104], [105]. The use of L929 to determine the cytotoxicity of medical devices following ISO 10993-5 is also applied in biomaterial researches on IOLs and other medical devices [99], [104], [105]. The use of the L929 cell line is preferred because it is established and obtained from recognized repositories (ISO 10993-5.5). In our case, since the virgin polymer itself did not attract cells, it would lead to a low viability value although it was proven to be non-cytotoxic. Alternatively, indirect cytocompatibility study is suggested in ISO 10993-5∶2009 by using conditioned medium to detect the release of toxic substances. Our data revealed that the RGD peptide-immobilized samples had cell viability values greater than 70%, indicating no cytotoxic potential by the ISO definition. In addition, the values were also comparable to those of the virgin polymer, which was proven to be non-toxic in clinical cases.

It is known that the surface hydrophilicity could affect cell adhesion behavior. If the surface is extremely hydrophobic, the adsorbed proteins will be denatured [106]. The receptors on the cellular membrane can hardly recognize the denatured proteins and thus, no cell can adhere. On the other hand, if the surface is extremely hydrophilic, no protein could be adsorbed, which leads again to no cell adhesion. Previous studies have shown that the optimized hydrophilicity for cell adhesion is in the range of 45° to 75° of aqueous contact angle [1], [106]. From our result (Fig. 10A), all the samples were located in the optimized range. The RGD and FITC-RGD peptide-adsorbed samples showed similar contact angles to those exhibited by the HA25 control sample, suggesting that the surface-adsorbed peptides were mostly removed during the wash process and had no effect on the contact angle. In addition, the oxygen plasma-treated samples became significantly more hydrophilic, which corresponds to the literature [80]. The phenomenon of RGD-grafted surfaces exhibiting even lower contact angles could be explained by the “Hydrophobic Recovery” effect of the material and hydrophilicity nature of the RGD peptide. Plasma-treated materials have been reported to have a “Hydrophobic Recovery” effect which a polymer partially restores the original hydrophobic surface to the extent that it adapts its composition to the interfacial force [80]. The mechanism of this effect is considered as the reorientation of the non-polar groups from the bulk to the surface or reorientation of polar groups from the surface to the bulk phase [107]. Since the grafting reaction conjugates peptides to the carboxyl group at the surface, the volume of the peptide would inhibit the reorientation of the carboxyl group into the bulk. In addition, the outer space occupied by the peptide may also be a hindrance to inhibit the reorientation of non-polar groups from the bulk to the surface. Overall, the grafting of peptides may lead to a reduced hydrophobic recovery effect [108]. On the other hand, the RGD peptide is mainly composed of hydrophilic amino acid residues. The FITC-RGD peptide-grafted surfaces, however, showed higher contact angles than the RGD-grafted surfaces and exhibited no significant difference compared to the plasma-treated surfaces, presumably because of the relative hydrophobic fluorescein moiety of the FITC-RGD peptide (Fig. 2). Therefore, RGD-grafted surfaces are more hydrophilic than plasma-treated or untreated HA25 surfaces with small contact angle differences.

In addition, the RGD peptides did not lose bioactivity even after multiple autoclaves. The wash step adapted 10 cycles of autoclaving providing an opportunity to examine not only the stability of the biological function of the peptide but also the robustness of the peptide-functionalized surface in harsh conditions. During the consecutive autoclaving process, the extraction of the FITC-RGD peptides occurred between the polymer and the deionized water. The peptide concentrations of the eluates were calculated by measuring the fluorescence. The concentration of the first few eluates may vary from different samples, but the last eluate showed as low as smaller than 10 µM in concentration (Fig. S2). This observation indicated the wash step removed most physically adsorbed RGD peptides and the potential free peptides dissociated in the physiological environment were too few to induce any adverse effect in the body, although the RGD peptide-based compound seems lack the maximum tolerated dose, as well as significant attributable toxicity [109]. Therefore, with the critical washing step, we control the bio-adhesive IOL surface without the risk of high concentration of RGD peptide released into the body's circulation.

## Conclusions

PCO has become a public health issue because of the rising incidence of age-related cataract and accompanied increase in IOL implantation. Ophthalmologists are awaiting a solution to prevent PCO. We propose a potential solution by functionalizing the surface of a conventional hydrophilic acrylic IOL material with the RGD peptide sequence to promote LEC adhesion. This strategy aims at recovering the synthetic intraocular lens to mimic the natural environment.

The surface functionalization process was accomplished by oxygen plasma treatment followed by conjugation, and the washing procedure consisted of consecutive water extraction in autoclave temperature. This new biomaterial exhibited similar LEC adhesion and morphology as well as EMT biomarker expression profiles compared to the hydrophobic material. The modification did not impede other properties (optical, mechanical, and cytotoxicity) required for an ophthalmic implant material.

Our pioneering *in vitro* study of the bio-mimicking strategy suggests that the LEC adhesion is promoted and this surface-functionalized IOL material exhibits no detectable effect toward EMT, which is the molecular cell biology mechanism of PCO. This hypothesis still needs to be examined by *in vivo* assays to validate the effectiveness of PCO prevention.

## Supporting Information

S1 Figure **Illustration of plasma, peptide-adsorbed, and peptide-graft samples from neat HA25 material.**
(TIF)

S2 Figure **Peptide concentrations of autoclave eluates of the FITC-RGD-immobilized disks.**
(TIF)

## References

[1] Bozukova D, Pagnoulle C, Jérôme R, Jérôme C (2010) Polymers in modern ophthalmic implants—Historical background and recent advances. Materials Science and Engineering: R: Reports 69:63–83.

[2] Agarwal A, Agarwal A, Jacob S (2011) Phacoemulsification: Jaypee Brothers, Medical Publishers.

[3] Werner LP, Legeais JM (1998) The materials for intraocular lenses. I – Rigid intraocular lenses. J Fr Ophtalmol, 1998 21:10 –524 21:

[4] Werner J-P, Legeais J-M (1999) The materials for intraocular lenses. II : silicone foldable intraocular lenses. J Fr Ophtalmol 22:10. 10365340

[5] Legeais J-M, Werner L, Werner L, Renard G (2001) The materials for intraocular lenses. Part III: acrylic foldable intraocular lenses. J Fr Ophtalmol 24:10. 11285449

[6] Niranjan Awasthi SG, Wagner BJ (2009) Posterior capsular opacification- a problem reduced but not yet eradicated. Arch Ophthalmol 127:8. 10.1001/archophthalmol.2009.3 19365040

[7] Juila M, Marcantonio GFJMV (1999) Cell biology of posterior capsular opacification. Eye 13:5. 10.1038/eye.1999.126 10627829

[8] Saika S (2004) Relationship between posterior capsule opacification and intraocular lens biocompatibility. Prog Retin Eye Res 23:283–305. 15177204 10.1016/j.preteyeres.2004.02.004

[9] Anderson JM (2001) Biological Responses to Materials. Annu Rev Mater Res 31:30.

[10] Spalton D (2012) Posterior capsule opacification: have we made a difference? British Journal of Ophthalmology: bjophthalmol-2012-302570. 10.1136/bjophthalmol-2012-302570 23077226

[11] Peng Q, Apple DJ, Visessook N, Werner L, Pandey SK, et al (2000) Surgical prevention of posterior capsule opacification. Part 2: Enhancement of cortical cleanup by focusing on hydrodissection. J Cataract Refract Surg 26:188–197. 10683786 10.1016/s0886-3350(99)00354-5

[12] Behar-Cohen F, El Aouni A, Le Rouic J-F, Parel J-M, Renard G, et al (2001) Iontophoresis: past and future. J Fr Ophtalmol 24:9. 11285450

[13] Nishi O, Nishi K, Wickström K (2000) Preventing lens epithelial cell migration using intraocular lenses with sharp rectangular edges. Journal of Cataract & Refractive Surgery 26:1543–1549. 11033405 10.1016/s0886-3350(00)00426-0

[14] Kohnen T, Fabian E, Gerl R, Hunold W, Hutz W, et al**.** (2008) Optic edge design as long-term factor for posterior capsular opacification rates. Ophthalmology 115:: 1308–1314, 1314 e1301–1303. 10.1016/j.ophtha.2008.01.002 18321585

[15] Hayashi K, Hayashi H (2005) Posterior capsule opacification in the presence of an intraocular lens with a sharp versus rounded optic edge. Ophthalmology 112:1550–1556. 16005976 10.1016/j.ophtha.2005.03.024

[16] Cheng JW, Wei RL, Cai JP, Xi GL, Zhu H, et al (2007) Efficacy of different intraocular lens materials and optic edge designs in preventing posterior capsular opacification: a meta-analysis. Am J Ophthalmol 143:428–436. 17224119 10.1016/j.ajo.2006.11.045

[17] James F. Boyce GSB, Spalton DJ, El-Osta AR (2002) Mathematical modeling of the forces between an intraocular lens and the capsule. J Cataract Refract Surg 28:7. 12388041 10.1016/s0886-3350(02)01490-6

[18] Nagamoto T, Fujiwara T (2003) Inhibition of lens epithelial cell migration at the intraocular lens optic edge. Journal of Cataract & Refractive Surgery 29:1605–1612. 12954314 10.1016/s0886-3350(03)00050-6

[19] Nixon DR, Apple DJ (2006) Evaluation of lens epithelial cell migration in vivo at the haptic-optic junction of a one-piece hydrophobic acrylic intraocular lens. Am J Ophthalmol 142:557–562. 17011844 10.1016/j.ajo.2006.05.049

[20] Kavoussi SC, Werner L, Fuller SR, Hill M, Burrow MK, et al (2011) Prevention of capsular bag opacification with a new hydrophilic acrylic disk-shaped intraocular lens. Journal of Cataract & Refractive Surgery 37:2194–2200. 22108114 10.1016/j.jcrs.2011.05.049

[21] Nagamoto T, Tanaka N, Fujiwara T (2009) Inhibition of posterior capsule opacification by a capsular adhesion–preventing ring. Archives of Ophthalmology 127:471–474. 19365027 10.1001/archophthalmol.2009.63

[22] Nishi O (2012) Other factors in PCO prevention. Journal of Cataract & Refractive Surgery 38:924–925. 22520328 10.1016/j.jcrs.2012.03.005

[23] Werner L (2008) Biocompatibility of intraocular lens materials. Current Opinion in Ophthalmology 19:: 41–49 10.1097/ICU.1090b1013e3282f20132. 10.1097/ICU.0b013e3282f20132 18090897

[24] Abela-Formanek C, Amon M, Schauersberger J, Kruger A, Nepp J, et al (2002) Results of hydrophilic acrylic, hydrophobic acrylic, and silicone intraocular lenses in uveitic eyes with cataract: Comparison to a control group. Journal of Cataract & Refractive Surgery 28:1141–1152. 12106722 10.1016/s0886-3350(02)01425-6

[25] Abela-Formanek C, Amon M, Schild G, Schauersberger J, Heinze G, et al (2002) Uveal and capsular biocompatibility of hydrophilic acrylic, hydrophobic acrylic, and silicone intraocular lenses. Journal of Cataract & Refractive Surgery 28:50–61. 11777710 10.1016/s0886-3350(01)01122-1

[26] Tognetto D, Toto L, Sanguinetti G, Cecchini P, Vattovani O, et al (2003) Lens epithelial cell reaction after implantation of different intraocular lens materials. Ophthalmology 110:1935–1941. 14522768 10.1016/S0161-6420(03)00736-X

[27] Bertrand V, Bozukova D, Svaldo Lanero T, Huang Y-S, Schol D, et al (2014) Biointerface multiparametric study of intraocular lens acrylic materials. Journal of Cataract & Refractive Surgery 40:1536–1544. 25135546 10.1016/j.jcrs.2014.01.035

[28] Linnola RJ WL, Pandey SK, Escobar-Gomez M, Znoiko SL, Apple DJ (2000) Adhesion of fibronectin, vitronectin, laminin, and collagen type IV to intraocular lens materials in pseudophakic human autopsy eyes. Part 1 histological sections. J Cataract Refract Surg 26:15. 10.1016/s0886-3350(00)00748-3 11134882

[29] Linnola RJ WL, Pandey SK, Escobar-Gomez M, Znoiko SL, Apple DJ (2000) Adhesion of fibronectin, vitronectin, laminin, and collagen type IV to intraocular lens materials in pseudophakic human autopsy eyes. Part 2 explanted intraocular lenses. J Cataract Refract Surg 26:12. 11134883 10.1016/s0886-3350(00)00747-1

[30] Linnola RJ, Sund M, Ylönen R, Pihlajaniemi T (2003) Adhesion of soluble fibronectin, vitronectin, and collagen type IV to intraocular lens materials. Journal of Cataract & Refractive Surgery 29:146–152. 12551682 10.1016/s0886-3350(02)01422-0

[31] Katayama Y KS, Yanagawa H, Tochikubo T (2007) The relationship between the adhesion characteristics of acrylic intraocular lens materials and posterior capsule opacification. Ophthalmic Res: 6. 10.1159/000108121 17851268

[32] Heatley CJ, Spalton DJ, Kumar A, Jose R, Boyce J, et al (2005) Comparison of posterior capsule opacification rates between hydrophilic and hydrophobic single-piece acrylic intraocular lenses. J Cataract Refract Surg 31:718–724. 15899448 10.1016/j.jcrs.2004.08.060

[33] Tatsuo Arita L-RL, Susan SR, Reddy VN (1990) Enhancement of Differentiation of Human Lens Epithelium in Tissue Culture by Changes in Cell-Substrate Adhesion. Investigative Ophthalmology & Visual Science, Vol 31, No 11 31:10. 2243005

[34] Linnola RJ (1997) Sandwich theory: bioactivity-based explanation for posterior capsule opacification. J Cataract Refract Surg 23:4. 9456413 10.1016/s0886-3350(97)80026-0

[35] Ruoslahti E PM (1987) New perspectives in cell adhesion: RGD and integrins. Science 238:7. 2821619 10.1126/science.2821619

[36] Dimitriya Bozukova CP, De Pauw-Gillet MC, Desbief S, Lazzaroni R, Ruth N, et al (2007) Improved performances of intraocular lenses by poly(ethylene glycol) chemical coatings. Biomacromolecules 8:9. 10.1021/bm0701649 17608449

[37] Lee JM, Dedhar S, Kalluri R, Thompson EW (2006) The epithelial–mesenchymal transition: new insights in signaling, development, and disease. The Journal of Cell Biology 172:973–981. 16567498 10.1083/jcb.200601018 PMC2063755

[38] Lee K, Nelson CM (2012) New insights into the regulation of epithelial-mesenchymal transition and tissue fibrosis. Int Rev Cell Mol Biol 294:171–221. 22364874 10.1016/B978-0-12-394305-7.00004-5

[39] Bozukova D, Pagnoulle C, Jerome C (2013) Biomechanical and optical properties of 2 new hydrophobic platforms for intraocular lenses. J Cataract Refract Surg 39:1404–1414. 23827766 10.1016/j.jcrs.2013.01.050

[40] Latz C, Migonney V, Pavon-Djavid G, Rieck P, Hartmann C, et al (2000) Inhibition of lens epithelial cell proliferation by substituted PMMA intraocular lenses. Graefes Arch Clin Exp Ophthalmol 238:696–700. 11011691 10.1007/s004170000153

[41] Wang GQ, Gu HQ, Peng XJ (2012) Study on the surface properties of surface modified silicone intraocular lenses. Int J Ophthalmol 5:84–87. 22553761 10.3980/j.issn.2222-3959.2012.01.17 PMC3340832

[42] Wang GQ, Gu HQ, Yuan JQ, Sun HM, Xu YS (2010) F-heparin modified intraocular lenses in Rhesus monkeys. Int J Ophthalmol 3:141–144. 22553538 10.3980/j.issn.2222-3959.2010.02.11 PMC3340764

[43] Liliana Werner J-ML, Nagel MD, Renard G (1999) Evaluation of teflon-coated intraocular lenses in an organ culture method. J Biomed Mater Res 46:8. 10.1002/(sici)1097-4636(19990905)46:3<347::aid-jbm6>3.0.co;2-m 10397991

[44] Lingli Li LL, Xu X, Nan K, Chen H (2011) Surface modification of intraocular lens material by poly(ethyleneglycol) methyl ether methacrylate via a plasma technique to influence posterior capsular opacification. Journal of Controlled Release 152:2. 22195868 10.1016/j.jconrel.2011.09.022

[45] Hyeon Il Lee MKK, Ko JH, Lee HJ, Wee WR, Lee JH (2007) The Efficacy of an Acrylic Intraocular Lens Surface Modified with Polyethylene Glycol in Posterior Capsular Opacification. J Korean Med Sci 22:6. 10.3346/jkms.2007.22.3.502 PMC2693645 17596661

[46] Sun CB, Teng WQ, Cui JT, Huang XJ, Yao K (2014) The effect of anti-TGF-beta2 antibody functionalized intraocular lens on lens epithelial cell migration and epithelial-mesenchymal transition. Colloids Surf B Biointerfaces 113:33–42. 24060928 10.1016/j.colsurfb.2013.08.024

[47] Amoozgar B, Fitzpatrick SD, Sheardown H (2013) Effect of anti-TGF-β2 surface modification of polydimethylsiloxane on lens epithelial cell markers of posterior capsule opacification. Journal of Bioactive and Compatible Polymers 28:637–651.

[48] Morarescu D, West-Mays JA, Sheardown HD (2010) Effect of delivery of MMP inhibitors from PDMS as a model IOL material on PCO markers. Biomaterials 31:2399–2407. 20022368 10.1016/j.biomaterials.2009.11.108 PMC2972668

[49] Huang X, Wang Y, Cai JP, Ma XY, Li Y, et al (2013) Sustained release of 5-fluorouracil from chitosan nanoparticles surface modified intra ocular lens to prevent posterior capsule opacification: an in vitro and in vivo study. J Ocul Pharmacol Ther 29:208–215. 23428176 10.1089/jop.2012.0184

[50] Porté-Durrieu MC LC, Villars F, Lefebvre F, Dutoya S, Guette A, et al (1999) Development of RGD peptides grafted onto silica surfaces: XPS characterization and human endothelial cell interactions. J Biomed Mater Res 46:8. 10.1002/(sici)1097-4636(19990905)46:3<368::aid-jbm9>3.0.co;2-8 10397994

[51] Das RKZO, Labrugère C, Oda R, Durrieu MC (2013) Influence of nanohelical shape and periodicity on stem cell fate. ACS Nano 7:11. 23451935 10.1021/nn4001325

[52] Céline Chollet SL, Brouillaud B, Labrugere C, Bareille R, Durrieu MC (2006) RGD Peptide Grafting onto Micro-patterned PET- Peptide Distribution Impact on Cell Attachment. Journal of Laser Micro/Nanoengineering 1:5.

[53] Bartouilh de Taillac L, Porté-Durrieu MC, Labrugère C, Bareille R, Amédée J, et al (2004) Grafting of RGD peptides to cellulose to enhance human osteoprogenitor cells adhesion and proliferation. Composites Science and Technology 64:827–837.

[54] Porte-Durrieu MC, Guillemot F, Pallu S, Labrugere C, Brouillaud B, et al (2004) Cyclo-(DfKRG) peptide grafting onto Ti-6Al-4V: physical characterization and interest towards human osteoprogenitor cells adhesion. Biomaterials 25:4837–4846. 15120531 10.1016/j.biomaterials.2003.11.037

[55] Poulin S, Durrieu MC, Polizu S, Yahia LH (2006) Bioactive molecules for biomimetic materials: Identification of RGD peptide sequences by TOF-S-SIMS analysis. Applied Surface Science 252:6738–6741.

[56] Chollet C, Chanseau C, Brouillaud B, Durrieu MC (2007) RGD peptides grafting onto poly(ethylene terephthalate) with well controlled densities. Biomol Eng 24:477–482. 17869172 10.1016/j.bioeng.2007.07.012

[57] Chollet C, Chanseau C, Remy M, Guignandon A, Bareille R, et al (2009) The effect of RGD density on osteoblast and endothelial cell behavior on RGD-grafted polyethylene terephthalate surfaces. Biomaterials 30:711–720. 19010529 10.1016/j.biomaterials.2008.10.033

[58] Chollet C, Lazare S, Guillemot F, Durrieu MC (2010) Impact of RGD micro-patterns on cell adhesion. Colloids Surf B Biointerfaces 75:107–114. 19775874 10.1016/j.colsurfb.2009.08.024

[59] Zouani OF, Chollet C, Guillotin B, Durrieu MC (2010) Differentiation of pre-osteoblast cells on poly(ethylene terephthalate) grafted with RGD and/or BMPs mimetic peptides. Biomaterials 31:8245–8253. 20667411 10.1016/j.biomaterials.2010.07.042

[60] Lei Y, Remy M, Labrugere C, Durrieu MC (2012) Peptide immobilization on polyethylene terephthalate surfaces to study specific endothelial cell adhesion, spreading and migration. J Mater Sci Mater Med 23:2761–2772. 22878726 10.1007/s10856-012-4736-x

[61] Lei Y, Zouani OF, Remy M, Ayela C, Durrieu MC (2012) Geometrical microfeature cues for directing tubulogenesis of endothelial cells. PLoS One 7:e41163. 22829923 10.1371/journal.pone.0041163 PMC3400641

[62] Cheng ZA, Zouani OF, Glinel K, Jonas AM, Durrieu MC (2013) Bioactive chemical nanopatterns impact human mesenchymal stem cell fate. Nano Lett 13:3923–3929. 23905702 10.1021/nl4020149

[63] Zouani OF, Kalisky J, Ibarboure E, Durrieu MC (2013) Effect of BMP-2 from matrices of different stiffnesses for the modulation of stem cell fate. Biomaterials 34:2157–2166. 23290467 10.1016/j.biomaterials.2012.12.007

[64] Zouani OF, Rami L, Lei Y, Durrieu MC (2013) Insights into the osteoblast precursor differentiation towards mature osteoblasts induced by continuous BMP-2 signaling. Biol Open 2:872–881. 24143273 10.1242/bio.20134986 PMC3773333

[65] Eisele G, Wick A, Eisele AC, Clement PM, Tonn J, et al (2014) Cilengitide treatment of newly diagnosed glioblastoma patients does not alter patterns of progression. J Neurooncol 117:141–145. 24442484 10.1007/s11060-014-1365-x

[66] Puleo DA, Bizios R (2009) Biological Interactions on Materials Surfaces: Understanding and Controlling Protein, Cell, and Tissue Responses: Springer.

[67] Carson AE, Barker TH (2009) Emerging concepts in engineering extracellular matrix variants for directing cell phenotype. Regen Med 4:593–600. 19580407 10.2217/rme.09.30 PMC2760348

[68] Daniele Tognetto LT, Ballone E, Ravalico G (2002) Biocompatibility of hydrophilic intraocular lenses. J Cataract Refract Surg 28:8. 11955905 10.1016/s0886-3350(01)01158-0

[69] Roberts C, Chen CS, Mrksich M, Martichonok V, Ingber DE, et al (1998) Using Mixed Self-Assembled Monolayers Presenting RGD and (EG)3OH Groups To Characterize Long-Term Attachment of Bovine Capillary Endothelial Cells to Surfaces. Journal of the American Chemical Society 120:6548–6555.

[70] Dick HB, Schwenn O, Krummenauer F, Krist R, Pfeiffer N (2000) Inflammation after sclerocorneal versus clear corneal tunnel phacoemulsification. Ophthalmology 107:241–247. 10690818 10.1016/s0161-6420(99)00082-2

[71] Fine IH, Hoffman RS, Packer M (2007) Profile of clear corneal cataract incisions demonstrated by ocular coherence tomography. Journal of Cataract & Refractive Surgery 33:94–97. 17189800 10.1016/j.jcrs.2006.09.016

[72] Chu PK, Chen JY, Wang LP, Huang N (2002) Plasma-surface modification of biomaterials. Materials Science and Engineering: R: Reports 36:64.

[73] Matsushima H, Iwamoto H, Mukai K, Obara Y (2006) Active oxygen processing for acrylic intraocular lenses to prevent posterior capsule opacification. J Cataract Refract Surg 32:1035–1040. 16814067 10.1016/j.jcrs.2006.02.042

[74] Lei Y ZO, Rami L, Chanseau C, Durrieu MC (2013) Modulation of lumen formation by microgeometrical bioactive cues and migration mode of actin machinery. Small 9:10. 10.1002/smll.201202410 23161822

[75] Zouani OF LY, Durrieu MC (2013) Pericytes, stem-cell-like cells, but not mesenchymal stem cells are recruited to support microvascular tube stabilization. Small 9:6. 10.1002/smll.201300124 23625793

[76] González-Chomón C, Concheiro A, Alvarez-Lorenzo C (2011) Drug-Eluting Intraocular Lenses. Materials 4:1927–1940. 28824115 10.3390/ma4111927 PMC5448846

[77] Sheardown H, Cooper SL (2003) Cells, Proteins and Materials: Festschrift in Honor of the 65th Birthday of Dr. John L. Brash: Taylor & Francis.

[78] Xu ZX, Li T, Zhong ZM, Zha DS, Wu SH, et al (2011) Amide-linkage formed between ammonia plasma treated poly(D,L-lactide acid) scaffolds and bio-peptides: enhancement of cell adhesion and osteogenic differentiation in vitro. Biopolymers 95:682–694. 21509742 10.1002/bip.21635

[79] De Giglio E, Cafagna D, Giangregorio M, Domingos M, Mattioli-Belmonte M, et al (2011) PHEMA-based thin hydrogel films for biomedical applications. Journal of Bioactive and Compatible Polymers 26:420–434.

[80] Lim H, Lee Y, Han S, Cho J, Kim K-J (2001) Surface treatment and characterization of PMMA, PHEMA, and PHPMA. Journal of Vacuum Science & Technology A: Vacuum, Surfaces, and Films 19:1490.

[81] Stevens JS, de Luca AC, Pelendritis M, Terenghi G, Downes S, et al (2013) Quantitative analysis of complex amino acids and RGD peptides by X-ray photoelectron spectroscopy (XPS). Surface and Interface Analysis 45:1238–1246.

[82] Briggs D (1999) Surface Analysis of Polymers by XPS and Static SIMS. Cambridge: Cambridge University Press.

[83] Goddard JM, Hotchkiss JH (2007) Polymer surface modification for the attachment of bioactive compounds. Progress in Polymer Science 32:698–725.

[84] Vasita RSIK, Katt DS (2008) Improved Biomaterials for Tissue Engineering Applications: Surface Modification of Polymers. Curr Top Med Chem 8:13. 10.2174/156802608783790893 18393896

[85] Ryu JJ, Park K, Kim HS, Jeong CM, Huh JB (2013) Effects of anodized titanium with Arg-Gly-Asp (RGD) peptide immobilized via chemical grafting or physical adsorption on bone cell adhesion and differentiation. Int J Oral Maxillofac Implants 28:963–972. 23869353 10.11607/jomi.2421

[86] Yang C, Cheng K, Weng W, Yang C (2009) Immobilization of RGD peptide on HA coating through a chemical bonding approach. J Mater Sci Mater Med 20:2349–2352. 19521750 10.1007/s10856-009-3794-1

[87] Luensmann D, Heynen M, Liu L, Sheardown H, Jones L (2009) Determination of albumin sorption to intraocular lenses by radiolabeling and confocal laser scanning microscopy. Journal of Cataract & Refractive Surgery 35:2000–2007. 19878835 10.1016/j.jcrs.2009.05.052

[88] Zeisberg M, Neilson EG (2009) Biomarkers for epithelial-mesenchymal transitions. J Clin Invest 119:1429–1437. 19487819 10.1172/JCI36183 PMC2689132

[89] Garcia CM, Kwon GP, Beebe DC (2006) alpha-Smooth muscle actin is constitutively expressed in the lens epithelial cells of several species. Exp Eye Res 83:999–1001. 16769052 10.1016/j.exer.2006.04.009 PMC2585415

[90] Power W, Neylan D, Collum L (1993) Morphological appearances of human lens epithelial cells in culture. Documenta Ophthalmologica 84:13–363 1993;84:10.1007/BF01215449 7512459

[91] Liu H, Wu L, Fu S, Hou Y, Liu P, et al (2009) Polylactide-glycoli acid and rapamycin coating intraocular lens prevent posterior capsular opacification in rabbit eyes. Graefes Arch Clin Exp Ophthalmol 247:801–807. 19066932 10.1007/s00417-008-1007-0

[92] Hongling Liu GF, Wu L, Fu S, Liu P, Yang W, et al (2010) The effects of rapamycin on lens epithelial cell proliferation, migration, and matrix formation- An in vitro study. 16:8. PMC2927374 20806034

[93] Pagnoulle C, Bozukova D, Gobin L, Bertrand V, Gillet-De Pauw MC (2012) Assessment of new-generation glistening-free hydrophobic acrylic intraocular lens material. J Cataract Refract Surg 38:1271–1277. 22727297 10.1016/j.jcrs.2012.02.041

[94] Kiefel H, Bondong S, Hazin J, Ridinger J, Schirmer U, et al (2012) L1CAM: a major driver for tumor cell invasion and motility. Cell Adh Migr 6:374–384. 22796939 10.4161/cam.20832 PMC3478260

[95] Bhattacharya SD, Mi Z, Talbot LJ, Guo H, Kuo PC (2012) Human mesenchymal stem cell and epithelial hepatic carcinoma cell lines in admixture: concurrent stimulation of cancer-associated fibroblasts and epithelial-to-mesenchymal transition markers. Surgery 152:449–454. 22938903 10.1016/j.surg.2012.06.011 PMC3432987

[96] Brown AC, Rowe JA, Barker TH (2011) Guiding epithelial cell phenotypes with engineered integrin-specific recombinant fibronectin fragments. Tissue Eng Part A 17:139–150. 20695776 10.1089/ten.tea.2010.0199 PMC3011912

[97] Zuk A, Hay ED (1994) Expression of beta 1 integrins changes during transformation of avian lens epithelium to mesenchyme in collagen gels. DEVELOPMENTAL DYNAMICS 201:16. 10.1002/aja.1002010409 7534501

[98] Prunier C, Howe PH (2005) Disabled-2 (Dab2) is required for transforming growth factor beta-induced epithelial to mesenchymal transition (EMT). J Biol Chem 280:17540–17548. 15734730 10.1074/jbc.M500974200

[99] Manju S, Kunnatheeri S (2010) Layer-by-Layer modification of poly (methyl methacrylate) intra ocular lens: drug delivery applications. Pharm Dev Technol 15:379–385. 19772379 10.3109/10837450903262025

[100] Werner L (2010) Glistenings and surface light scattering in intraocular lenses. Journal of cataract and refractive surgery 36:1398–1420. 20656166 10.1016/j.jcrs.2010.06.003

[101] Yan D, Jones J, Yuan XY, Xu XH, Sheng J, et al (2013) Plasma treatment of electrospun PCL random nanofiber meshes (NFMs) for biological property improvement. J Biomed Mater Res A 101:963–972. 22965926 10.1002/jbm.a.34398

[102] Kanokpanont S, Damrongsakkul S, Ratanavaraporn J, Aramwit P (2013) Physico-chemical properties and efficacy of silk fibroin fabric coated with different waxes as wound dressing. Int J Biol Macromol 55:88–97. 23313451 10.1016/j.ijbiomac.2013.01.003

[103] Susanne Krag TTA (2003) Mechanical Properties of the Human Posterior Lens Capsule. Invest Ophthalmol Vis Sci 44:6. 10.1167/iovs.02-0096 12556400

[104] Wadajkar AS, Ahn C, Nguyen KT, Zhu Q, Komabayashi T (2014) In Vitro Cytotoxicity Evaluation of Four Vital Pulp Therapy Materials on L929 Fibroblasts. ISRN Dentistry 2014:4. 10.1155/2014/191068 PMC3958681 24724032

[105] Wright A, Mowrey-McKee M (2005) Comparative cytotoxicity potential of soft contact lens care products. Cutan Ocul Toxicol 24:53–64. 17040888 10.1081/CUS-200046191

[106] Guelcher SA, Hollinger J (2005) An Introduction to Biomaterials: Taylor Francis Group.

[107] Kim J, Chaudhury MK, Owen MJ (2000) Hydrophobic Recovery of Polydimethylsiloxane Elastomer Exposed to Partial Electrical Discharge. Journal of Colloid and Interface Science 226:231–236. 10.1016/j.jcis.2005.06.068 16055136

[108] Bodas D, Khan-Malek C (2007) Hydrophilization and hydrophobic recovery of PDMS by oxygen plasma and chemical treatment—An SEM investigation. Sensors and Actuators B: Chemical 123:368–373.

[109] Reardon DA, Neyns B, Weller M, Tonn JC, Nabors LB, et al (2011) Cilengitide: an RGD pentapeptide alphanubeta3 and alphanubeta5 integrin inhibitor in development for glioblastoma and other malignancies. Future Oncol 7:339–354. 21417900 10.2217/fon.11.8

