# Supplementary figures and images for: RGD Surface Functionalization of the Hydrophilic Acrylic Intraocular Lens Material to Control Posterior Capsular Opacification
Source: PLoS One. 2014 Dec 11;9(12):e114973. doi: 10.1371/journal.pone.0114973 (PMC4263720; doi:10.1371/journal.pone.0114973)

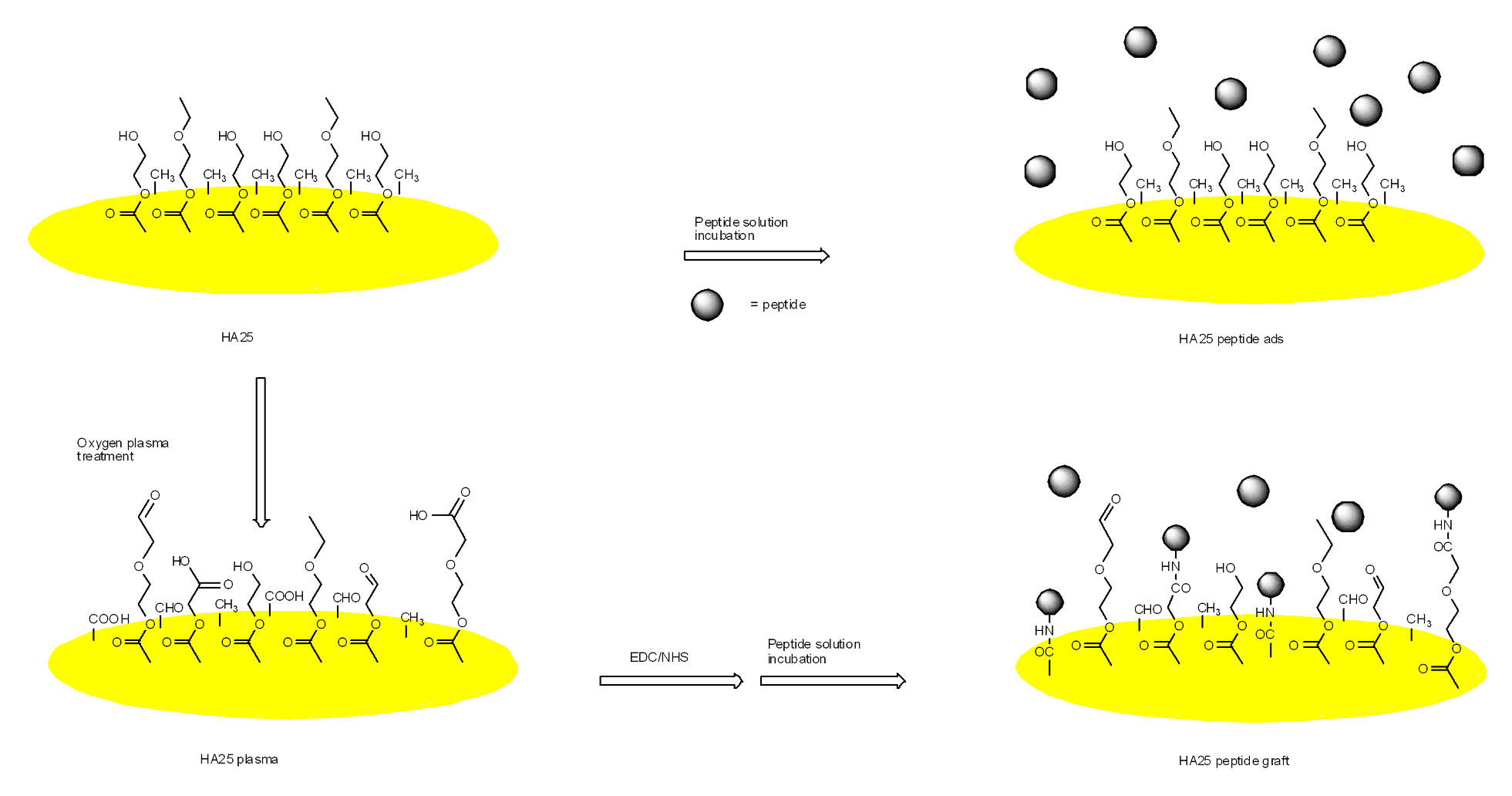

Supplement: S1 Figure — Illustration of plasma, peptide-adsorbed, and peptide-graft samples from neat HA25 material. (TIF) [file pone.0114973.s001.tif]

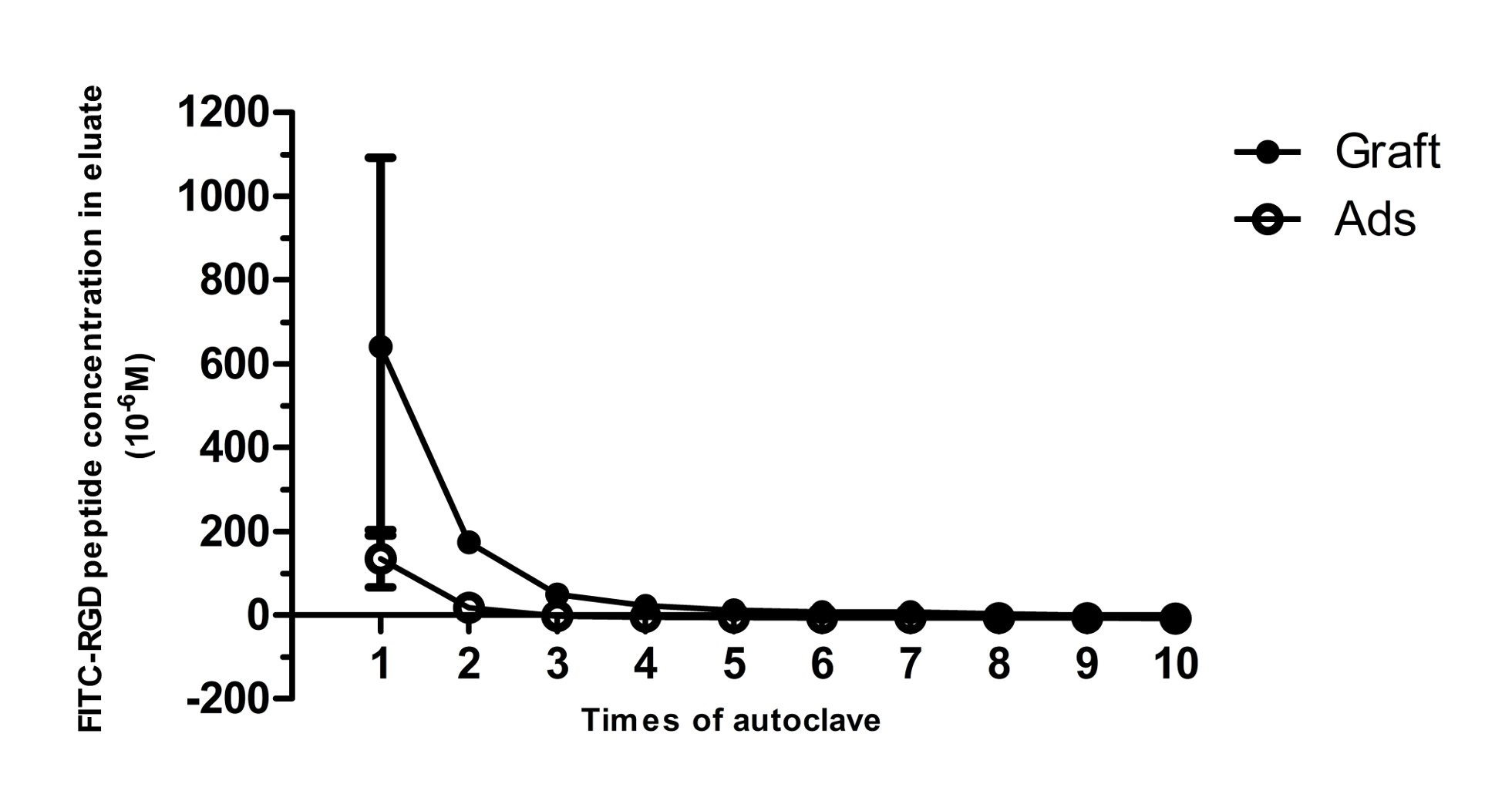

Supplement: S2 Figure — Peptide concentrations of autoclave eluates of the FITC-RGD-immobilized disks. (TIF) [file pone.0114973.s002.tif]
